# Analysis of the MODIST Sequence for Selective Proton–Proton Recoupling

**DOI:** 10.1021/acs.jpca.4c05102

**Published:** 2024-12-23

**Authors:** Evgeny Nimerovsky, Marianna Stampolaki, Abel Cherian Varkey, Stefan Becker, Loren B. Andreas

**Affiliations:** Department of NMR based Structural Biology, Max Planck Institute for Multidisciplinary Sciences, Am Faßberg 11, Göttingen 37077, Germany

## Abstract

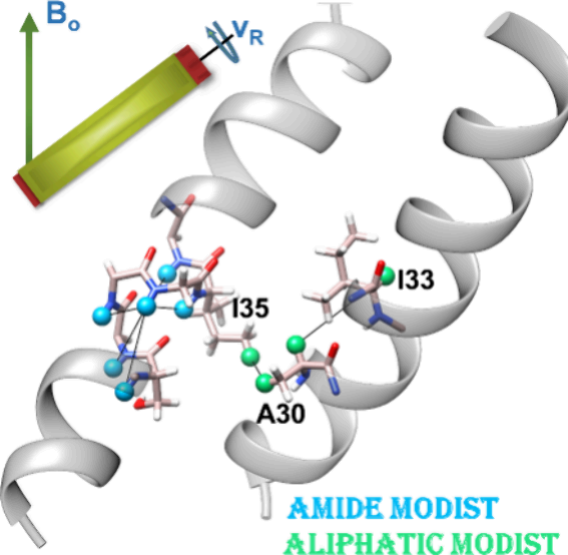

Theoretical and simulated analyses of selective homonuclear dipolar recoupling sequences serve as primary tools for understanding and determining the robustness of these sequences under various conditions. In this article, we investigate the recently proposed first-order dipolar recoupling sequence known as MODIST (Modest Offset Difference Internuclear Selective Transfer). We evaluate the MODIST transfer efficiency, assessing its dependence on rf-field strengths and the number of simulated spins, extending up to 10 spins. This helps to identify conditions that enhance polarization transfer among spins that are nearby in frequency, particularly among aliphatic protons. The exploration uncovers a novel effect for first-order selective recoupling sequences that we term “facilitated dipolar recoupling”. This effect amplifies the recoupled dipolar interaction between distant spins due to the presence of additional strongly dipolar-coupled spins. Unlike the third spin-assisted recoupling mechanism, facilitated dipolar recoupling only requires a coupling to one of the two distant spins of interest. Experimental demonstration of MODIST, including at different rf-field strengths, was carried out with the membrane protein influenza A M2 in lipid bilayers using 55 kHz magic-angle spinning (MAS). Reducing MODIST rf-field strength by a factor of 2 unveils possibilities for detecting Hα–Hα and H^Meth^–H^Meth^ correlations with a 3D (H)C(H)(H)CH experiment under fast MAS rates, all achievable without specific spin labeling.

## Introduction

Theoretical and numerical investigations of various homonuclear dipolar recoupling sequences^1−12^ aim to identify the conditions that ensure maximal efficiency of these sequences. These investigations are pivotal for acquiring and analyzing spectra that correlate nearby spins, a fundamental approach for amino acid assignments, and elucidating the structures and dynamic processes of biological macromolecules using proton-detected^13,14^ magic angle spinning^15,16^ (MAS) NMR.^14,17−25^

While carbon–carbon recoupling sequences have been a mainstay of MAS NMR experiments,^26−35^ proton–proton recoupling sequences are increasingly recognized to offer potential in detecting long-distance correlations. These correlations can be instrumental in determining the secondary and tertiary structures of folded proteins.^14,23,31,36−39^

Theoretical and numerical tools^40−44^ contribute significantly toward understanding the preferential excitation of short-distance correlations over long-distance correlations when employing first-order broad-band recoupling sequences^45−52^ in multidimensional experiments with uniformly labeled samples. In these scenarios, short-distance correlations, typically having the strongest dipolar coupling, dominate the resulting spectra, termed the dipolar truncation effect.^53−55^ Several strategies have emerged to tackle the dipolar truncation effect. Specific spin labeling,^56−62^ represents one solution, enabling the detection of long-distance correlations among backbone protons,^63,64^ as well as between side-chain protons.^32,64−68^ For samples where specific spin labeling is challenging (e.g., membrane proteins),^69^ various specialized recoupling sequences have been proposed, including spin-diffusion type sequences^70−76^ and second-order sequences.^45,46,77−83^ Additionally, selective dipolar recoupling sequences^18,27,46^ have been developed to address the dipolar truncation effect. Depending on the selectivity mechanism, these sequences are characterized as first-order band-selective,^84−87^ first-order frequency-selective^88−92^ and second-order band-selective methods.^93^

Theoretical and numerical investigations of broad-band^3,27,47,53,94,95^ and selective recoupling sequences^96−100^ have contributed significantly to the understanding of multi-spin dynamics, which led to development of novel selective recoupling sequences. With the introduction of a third spin into the theoretical picture comes the possibility for different transfer mechanisms. In addition to direct transfer, relayed transfer^53^ occurs due to first-order recoupling. In this case, polarization diffuses through intermediate spins, with dipolar truncation effects dictating flow of polarization through the stronger couplings. For example, in a homonuclear three-spin system, and first-order recoupling, the transferred signal between a distant spin pair can occur via a third-spin located between the distant pair. By contrast, third-spin assisted recoupling (TSAR)^101^ is a second-order effect. The third, assisting spin, does not receive polarization, but rather assists transfer via dipolar couplings to the other two spins. The third spin is often a proton, since a higher gamma nucleus is beneficial for generating a sizable second-order effect. Average Hamiltonian theory^42^ (AHT) adeptly describes the above phenomena.

A potential limitation of AHT is that only a few spins are typically considered in analytical derivations, from which conclusions are extended to systems with a higher number of relevant spins. This limitation can be anticipated to be particularly relevant for proton spins, which occur at high densities in proteins. While considering that just a few spins has been successful in identifying useful pulse sequences, simulations involving a greater number of spins can provide additional insight.

In this article, we use AHT and numerical simulations^102^ to investigate MODIST (Modest Offset Difference Internuclear Selective Transfer) for proton–proton recoupling. Our simulations explore the MODIST signal’s dependency on the number of spins (up to ten), offset differences and the applied rf-field strengths. While MODIST with the previously reported rf-field strength of 0.5ν_*R*_ (ν_*R*_ is a MAS rate in kHz units) demonstrated high efficiency for selective excitation of amide–amide correlations,^85^ this value (0.5ν_*R*_) does not have any special significance, unlike resonance conditions.^50,103−105^ For MODIST, changing the rf-field strength alters both the selectivity and the rate of polarization transfer. While this dependence does not define any specific optimal rf-field value, we used numerical simulations to investigate MODIST with three different rf-field strengths (flip angles) – 0.25ν_*R*_ (22.5°), 0.5ν_*R*_ (45°) and 1.12ν_*R*_ (101°) – and identified the experimental conditions under which each may offer higher efficiency, depending on selectivity.

Furthermore, we uncover an intriguing effect that we call ‘facilitated dipolar recoupling’ (FDR). In a two-spin system, polarization transfer using MODIST can be inefficient due to a large offset difference between the spin pair. However, in a multiple-spin system, improved transfer is observed when at least one of the two spins is strongly dipolar-coupled to additional spins. Notably, this effect differs from third-spin assisted recoupling, as it does not necessitate the placement of the additional spins between (having dipolar couplings with both) the spins to be recoupled.

We verified the MODIST performance at 55.555 kHz MAS for different rf-field strengths and magnetic fields, with measurements using membrane protein samples. Particularly, MODIST with an rf-field strength of 0.25ν_*R*_ enables the observation of additional correlations between aliphatic protons that are nearby in the spectrum, for example, Hα-Hα correlations, without the need for specific labeling.

## Average Hamiltonian Theory and Numerical Simulation

**Figure 1:**
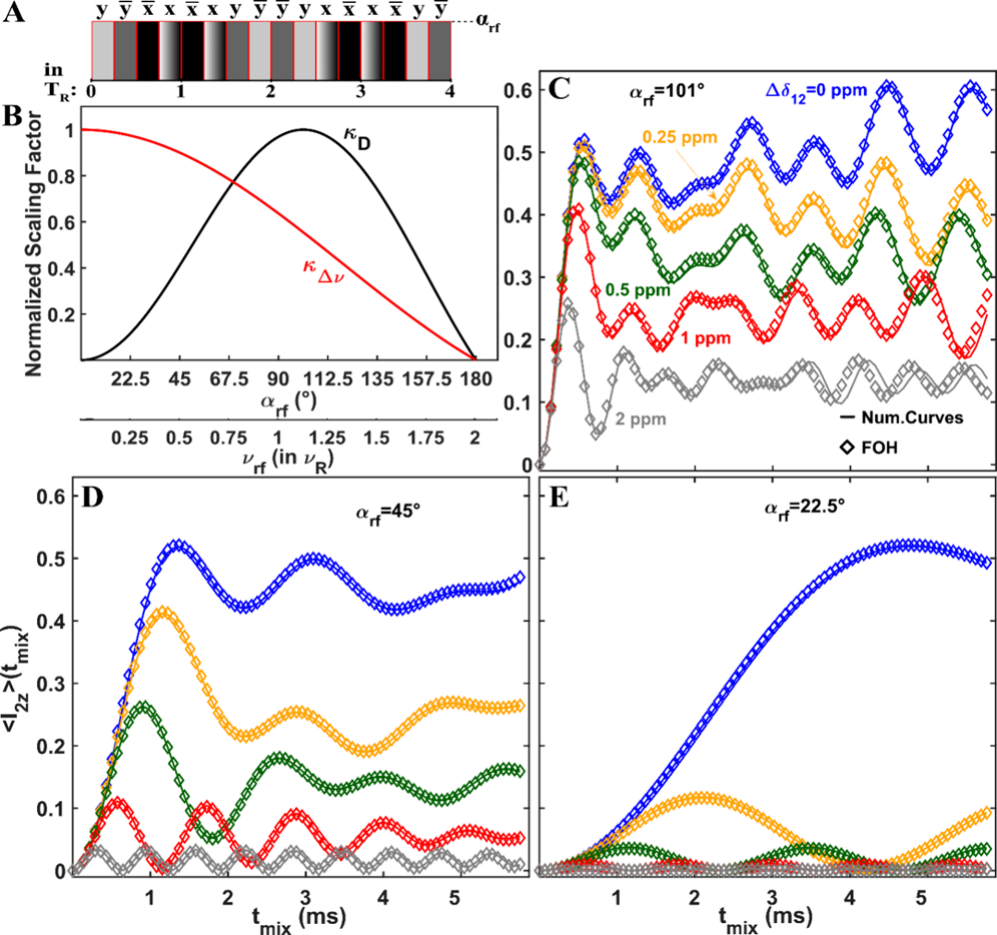
Theoretical and numerical analysis of MODIST for a two-spin system. (A) The MODIST pulse sequence, consisting of 16 pulses each with duration 0.25*T*_R_ and a flip angle of α_*rf*_. The phase is changed for each pulse according to *yy̅x̅xx̅xy̅yy̅yxx̅xx̅yy̅*, and the sequence is repeated to reach the required mixing time. (B) The normalized dipolar (*k*_*D*_) and isotropic chemical shift (*k*_Δν_) scaling factors as a function of α_*rf*_ (ν_*rf*_). (C)-(E) The FOH (diamonds, eq 1)) and numerical (lines) transferred signals as a function of mixing time and offset difference as indicated in (C), from 0 to 2 ppm. The chemical shift of spin 1 was kept constant (8.5 ppm) and the chemical shift of the spin 2 was a variable. All simulations used 7 kHz dipolar coupling (2.6 Å distance), 55 kHz MAS and 850 MHz ^1^H Larmor frequency.

The MODIST pulse sequence^85^ (depicted in Figure 1A) is constructed from a repeating set of pulses of constant amplitude and having the following phases: *yy̅x̅xx̅xy̅yy̅yxx̅xx̅yy̅*. Each single pulse has a duration of 0.25*T*_*R*_ and a flip angle α_*rf*_ = 2πν_*rf*_(0.25*T*_*R*_) = 0.5πν_*rf*_*T*_*R*_. Here, *T*_*R*_ represents the rotor period (1/ν_*R*_), while ν_*rf*_ indicates the nutation frequency due to the applied rf-field. The total duration of the block is 4*T*_*R*_, extendable in length through repetition *N* times. Overall, MODIST, which includes a jump-return element,^106,107^ belongs to the family of sequences^85,88,108^ with at least C2 symmetry.^109^ While MODIST has the structure of a C4 sequence, this symmetry is not unique, and phase cycling based on C2 symmetry (Figure S8 in ref (85)) provides a transfer efficiency similar to that of C4.

For a system of two-spins, average Hamiltonian theory (AHT) provides a useful framework for the derivation of analytical solutions to the equation of motion.^44^ The main behavior of the sequence can often be appreciated from the first-order Hamiltonian (FOH), where the total Hamiltonian is initially transformed into one of the possible interaction frames,^5^ which is a tilted rf-field frame in our case.^110^ The derivation of the FOH for MODIST is detailed in the Supporting Information (SI), while in the main text we present the final result. The theoretical transferred signal between spins *I*_1_ and *I*_2_ is described by the following equation:
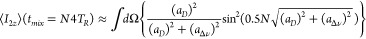
1where *a*_*D*_ = −*k*_*D*_*sin*^2^(β) *cos* (2γ)(ϖ_*D*_4*T*_*R*_), *a*_Δν_ = *k*_Δν_(Δν_12_4*T*_*R*_),

ϖ_*D*_ = ν_*D*_*sin*^2^(β) *cos* (2γ) and Δν_12_ = ν_1_ – ν_2_.

The values ν_1_, ν_2_ are the isotropic chemical shifts of the spin 1 and 2, respectively;  is a dipolar coupling value; *N* is the number of times that the basic MODIST block is repeated. The integration over orientation (Ω) indicates the powder averaging with Euler angles, (α, β, γ).^110^ The values, *k*_*D*_ and *k*_Δν_, represent the scaling factors associated with dipolar and isotropic chemical shift terms:
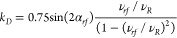
2A
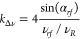
2Bwhere α_*rf*_ = 0.5πν_*rf*_*T*_*R*_.These scaling factors determine the extent of the influence of the recoupled interactions. Specifically, *k*_*D*_ governs the rate of the polarization transfer between dipolar coupled spin pairs, while *k*_Δν_ controls the selectivity of the sequence with respect to frequency difference. Figure 1B illustrates the relationship between normalized *k*_*D*_ (in black) and *k*_Δν_ (in red) values concerning the flip angle of the single pulse (α_*rf*_) within MODIST. *k*_*D*_ demonstrates zero values at α_*rf*_ = 0° and α_*rf*_ = 180°, with a global maximum at α_*rf*_ = 101°. On other hand, *k*_Δν_ attains its maximum value at 0° and decreases only slightly at 22.5° of α_*rf*_, then gradually decreases to zero at α_*rf*_ = 180°. In light of this plot, we consider three flip angle conditions: 101° (maximized *k*_*D*_), 22.5° (maximized *k*_Δν_ and a nonzero *k*_*D*_) and 45° (was previously introduced^85^).

To evaluate the transfer efficiency of MODIST using these three flip angles, we initially examine the simulated signal transfer for a two-spin system. Figure 1C-E presents comparisons between the theoretical (solid, eq 1)) and numerical (diamonds) transferred signals at different offset difference values (Δδ_12_, ppm) and flip angle settings of α_*rf*_ = 101° (C), α_*rf*_ = 45° (D) and α_*rf*_ = 22.5° (E). Overall, we observe good agreement between the numerical simulations and the solution to the first-order average Hamiltonian.

For an isolated two-spin system, high transfer efficiency is observed irrespective of flip angle provided the spins have the same frequency (Δδ_12_ = 0). The time required to reach the first maximum reflects the different scaling factors (Figure 1B). As expected from the first-order average Hamiltonian, MODIST demonstrates the highest selectivity with α_*rf*_ = 22.5° (Figure 1E), while with α_*rf*_ = 101° (Figure 1C), it exhibits the least selectivity among all three.

According to simulations for the two-spin system, the second flip angle (at 22.5°) is inefficient, compared to the other two, as MODIST with this angle yields a very narrow width of selective transfer, Δ*f*_*MODIST*_. Δ*f*_*MODIST*_ refers to the offset difference at which the transferred signal reaches 50% of the maximal transfer concerning the signal with zero offset values. To estimate Δ*f*_*MODIST*_, we selected the transferred signals at the first global maximum. With a 101° flip angle and 2.6 Å distance, Δ*f*_*MODIST*_ is approximately 1700 Hz (2 ppm at an 850 MHz spectrometer), while with 22.5°, it is only 60 Hz (0.07 ppm at an 850 MHz spectrometer).

**Figure 2:**
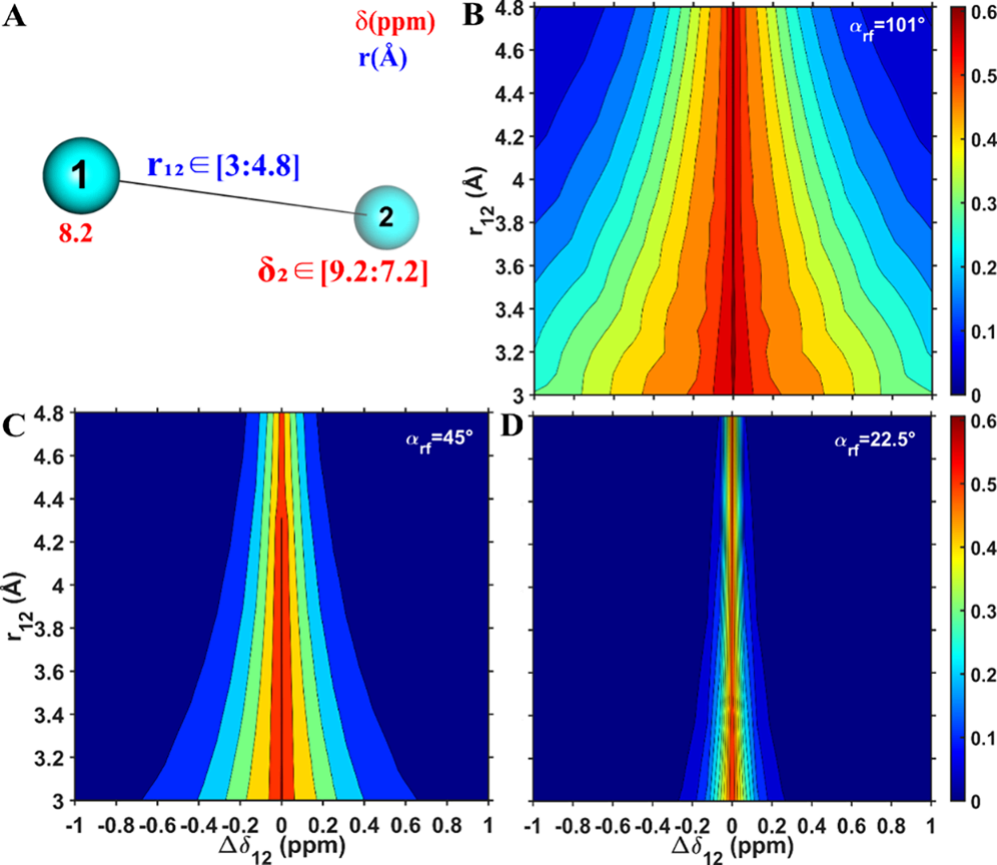
Numerical MODIST transfer efficiency as a function of offset difference (in ppm, *x*-axis) and distance (in Å, *y*-axis) for the two-spin system shown in (A). Three different flip angles are considered: 101° (B), 45° (C) and 22.5° (D). The isotropic chemical shift of spin 1 was fixed, and the isotropic chemical shift of spin 2 was varied. All simulations were performed using 55 kHz MAS, an 8.3 ppm carrier frequency, and an 850 MHz ^1^H Larmor frequency.

**Figure 3:**
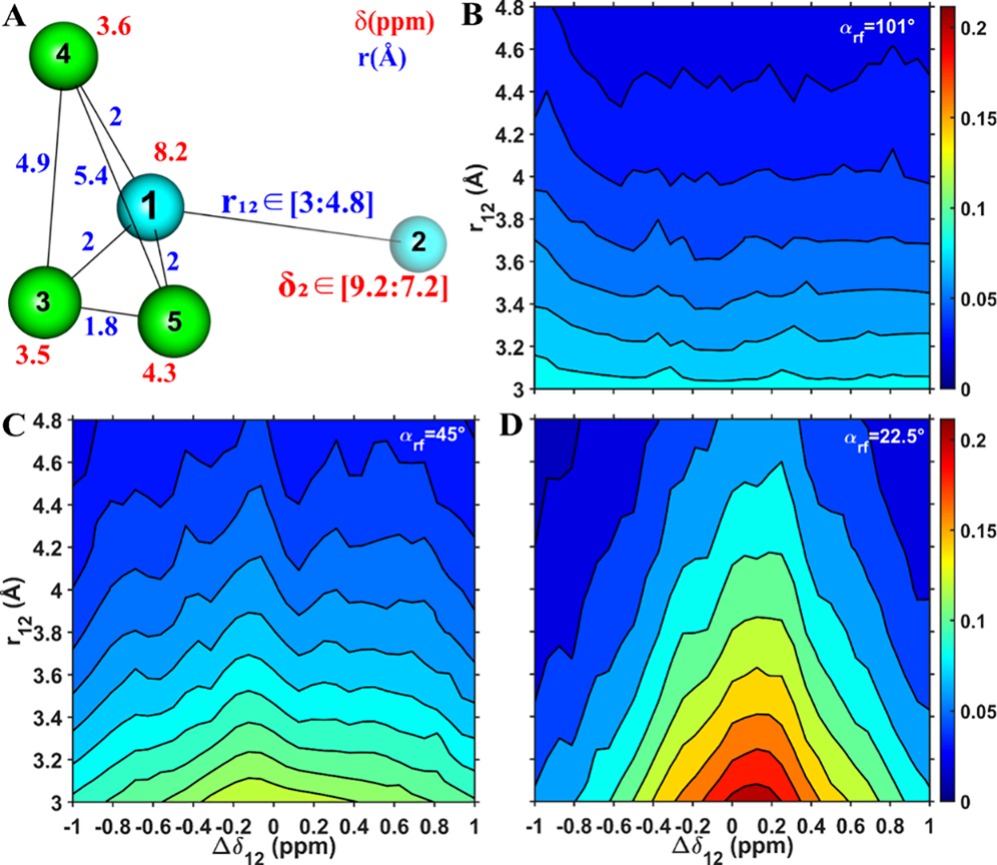
Numerical MODIST transfer efficiency as a function of offset difference (in ppm, *x*-axis) and distance (in Å, *y*-axis) was conducted for the five-spin system shown in (A). Distance-dependent transfer is considered for transfer from spin 1 to spin 2, which represent amide protons, using three different flip angles: 101° (B), 45° (C) and 22.5° (D). (A) schematically displays the simulated spin system: two amide protons (cyan spheres: 1 and 2) and three aliphatic (green spheres: 3–5). The isotropic chemical shifts of spins 1 and 3–5 were fixed (as shown in red in A), while the isotropic chemical shift of spin 2 was varied. The distance between spins 1 and 2 was varied, while the rest of the distances were fixed (as shown in A). All simulations were performed using 55 kHz MAS, an 8.3 ppm carrier frequency, and an 850 MHz ^1^H Larmor frequency.

Further investigations into the dependence of the MODIST transfer efficiency on flip angles, offset differences and the distances between a spin pair are shown in the following two figures for a two-spin system (Figure 2) and a five-spin system (Figure 3). The spin systems are illustrated in panel A of the respective figures, and in each case, the distance dependence is shown for a pair of spins that represent amide protons.

For the two-spin system (Figure 2), with zero offset difference, all three flip angles (101° - 2B; 45° - 2C and 22.5° - 2D) provide similar transfer efficiency regardless of the distance between the simulated spins. However, the Δ*f*_*MODIST*_ for all three flip angles depends on the distance, decreasing with an increase in the distance. At the longest simulated distance of 4.8 Å, Δ*f*_*MODIST*_ is 0.25, 0.06, and 0.02 ppm for the 101°, 45° and 22.5° flip angles, respectively. Such small Δ*f*_*MODIST*_ values for a two-spin system suggests impracticality in using MODIST with 45° and 22.5° flip angles for detection of distant proton–proton correlations.

With the addition of three strongly dipolar-coupled aliphatic spins (Figure 3), the transfer efficiencies for all three flip angles are reduced (compared to the two-spin system, Figure 2), and become dependent on the distance. While at a 3 Å distance and zero offset difference, MODIST provides 8%, 13% and 21% transfer efficiency for 101°, 45° and 22.5° flip angles, respectively, at a 4.8 Å distance, these values are reduced to 2.5%, 4.3% and 7.2%, respectively. Interestingly, MODIST with a 22.5° flip angle shows higher transfer efficiency, compared to the other two flip angles. While the transferred signal is truncated in the presence of a third strongly bonded spin for MODIST with 101° and 45° flip angles (Figure S1B-C in the SI), it is enhanced with a 22.5° flip angle (Figure S1D in the SI) when one considers reasonable offset differences.

The presence of the strongly dipolar-coupled aliphatic spins clearly increases Δ*f*_*MODIST*_ for all three flip angles, thereby broadening and enhancing MODIST transfer efficiency for the proton–proton correlations of interest, here representing amide–amide transfer. For the five-spin system, the dependence of Δ*f*_*MODIST*_ on distance decreases. In this simulation, Δ*f*_*MODIST*_ becomes roughly 6, 1.2, and 0.6 ppm, for the 101°, 45° and 22.5° flip angles, respectively.

However, when the additional dipolar-coupled spins weakly interact with the initially polarized spin, MODIST becomes overly selective, similar to what is observed in a two-spin system (Figure 2). As a result, MODIST is only efficient for recoupling proton–proton correlations with high spin density and strong dipolar interactions. The inefficiency of MODIST in recoupling carbon spins with weak dipolar coupling values motivated us to develop another selective recoupling method, called GODIST.^108^

It is worth highlighting that the additional aliphatic spins were introduced in the simulation without couplings to the amide spin number 2, while remaining close to the spin number 1 (∼2.0 Å). This configuration of the spin system eliminates the possibility of transfer via the TSAR mechanism^101^ and suggests the consideration of another effect, which we call facilitated dipolar recoupling (FDR). In FDR, additional spins play a passive role–improving transfer efficiency for spins with nonzero offset differences–while in TSAR, additional spins play an active role by connecting distant spins via dipolar couplings to both.

To investigate FDR in detail, particularly whether it depends solely on first-order terms or whether it includes higher-order terms, the numerical transferred signals were compared with FOH curves in multispin systems. The calculated FOH for a two-spin system (eq (S37) in the SI) can be extended to an n-spin system,^27^ which is defined by the following equation.:
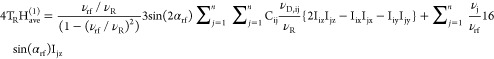
3where ν_j_ – offset of spin j in Hz units; ν_D, ij_ – the dipolar coupling between spins i and j in Hz units. C_ij_ is the spatial part of the dipolar interaction, which depends on three Euler angles (α, β, γ) and two angles (θ_ij_, ϕ_ij_) that define the orientation between different dipolar principal axis systems:^111^
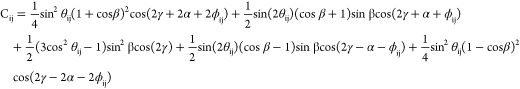
4

**Figure 4:**
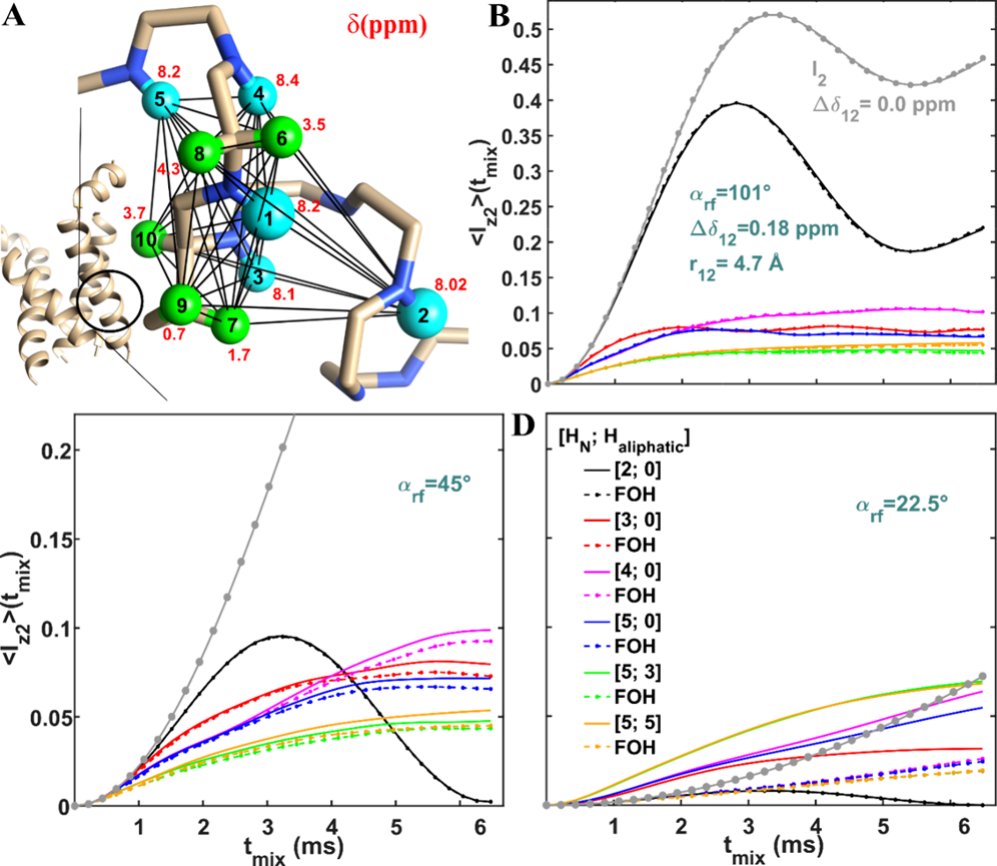
Numerical (solid) and FOH (dashed lines with points) analysis of MODIST was conducted for systems comprising up to ten spins, using three different flip angles: 101° (B), 45° (C) and 22.5° (D). The transferred signal as a function of mixing time and the number of simulated spins [amide spins; aliphatic spins]: black – [2;0]; red – [3;0]; magenta – [4;0]; blue – [5;0]; green – [5;3]; orange – [5;5]. The gray solid lines and dashed lines with points represent the transferred signals for a two-spin system with zero offset difference. (A) schematically displays the simulated spin system: five amide protons (cyan spheres: 1–5) and five aliphatic protons (green spheres: 6–10). The structure of influenza A M2 (in A) is illustrated from the geometry found in pdb 2N70 of Andreas et al.^112^ In all simulations, the initial signal was on spin 1 and the transferred signal was measured on spin 2. The distance and the isotropic chemical shift difference between spin 1 and spin 2 were 4.7 Å and 0.18 ppm, respectively. The isotropic chemical shifts of all spins are displayed in red (in ppm units). The distances between pairs of spins are shown in “Simulations” in Table S6 in the SI. The spatial coordinates of the ten spins were taken from the helical structure of Influenza A M2. The dipolar coupling values and the angles between all spin pairs (i,j) were calculated according to these coordinates. All simulations used 55 kHz MAS, 8.3 ppm carrier frequency and 850 MHz ^1^H Larmor frequency.

Figure 4 compares the calculated signal transfer using either numerical (solid lines) or FOH (dashed lines with points) simulations. The curves, in each case, are for transfer from spin 1 to spin 2 in a multiple spin system, ranging from two to ten-spins. The simulated system is schematically illustrated in Figure 4A.

In general, we consider a simulated spin system that represents a nondeuterated protein sample with high amide and aliphatic spin densities. The spatial coordinates of ten spins (five amide and five aliphatic proton spins) were selected from the helical structure of Influenza A M2 (located at the S31–I32–I33-G34-I35-L36 residues). In the following simulations, the calculated signal transfer is from one amide proton to another (labeled as spins 1 and 2 in Figure 4A, with a distance of 4.7 Å) and occurs in the presence of up to eight additional strongly dipolar-coupled spins (spins 3–10). These spins are located closer to spin 1 than to spin 2. Table S6 (in the SI) summarizes all distances (r_ij_) and orientations (θ_ij_, ϕ_ij_) between each pair of spins (i,j).

For a two-spin system with a 0.18 ppm offset difference, a 101° flip angle (Figure 4B, black) results in 40% transfer efficiency. With a 45° flip angle (Figure 4C, black), the curve reaches 10% intensity, but drops to zero at longer mixing times. For a 22.5° flip angle (Figure 4D, black), the transferred signal is negligible.

Several interesting observations can be made from the numerical (solid lines) and FOH (dashed lines with points) curves when additional spins are included in the simulation. For the 101° flip angle (Figure 4B), there is a full agreement between the numerical and FOH curves. For the 45° flip angle (Figure 4C), the solid and dashed curves begin to deviate at longer mixing times. For the 22.5° flip angle (Figure 4D), the agreement between numerical and FOH curves is observed only for the two-spin system (black). For the other simulations in this figure, FOH predicts transfer efficiencies at least three times smaller than the numerical simulations. For 22.5° flip angle, the additional spins (amide and aliphatic) increase the transferred signal compared to the signal in two-spin simulations with zero offset difference (gray) and 0.18 ppm offset difference (black).

Notably, the simulated performance of MODIST with different flip angles changes when more spins are included in the system. For 101° (Figure 4B), the transfer efficiency is reduced due to dipolar truncation,^55^ as eight additional spins are located close to the spin with initial polarization (spin 1). For the 45° flip angle (Figure 4C), increasing the system to three (red) or four (blue) spins “stabilizes” the transfer efficiency, allowing a nonzero signal throughout the entire mixing time. Increasing the simulated system to ten-spins reduces the overall transfer efficiency (blue, green and orange solid lines). For the 22.5° flip angle (Figure 4D), increasing the number of spins not only stabilizes the transferred signal, but also improves the transfer efficiency compared to the initial two-spin simulations (black solid). For the ten-spin system simulations, MODIST with 101° and 45° flip angles yield similar transfer efficiencies (Figure 4B-C, orange solid line) of 5.5%, while the 22.5° shows a slightly better performance at 6.8% (Figure 4D, orange solid line).

**Figure 5:**
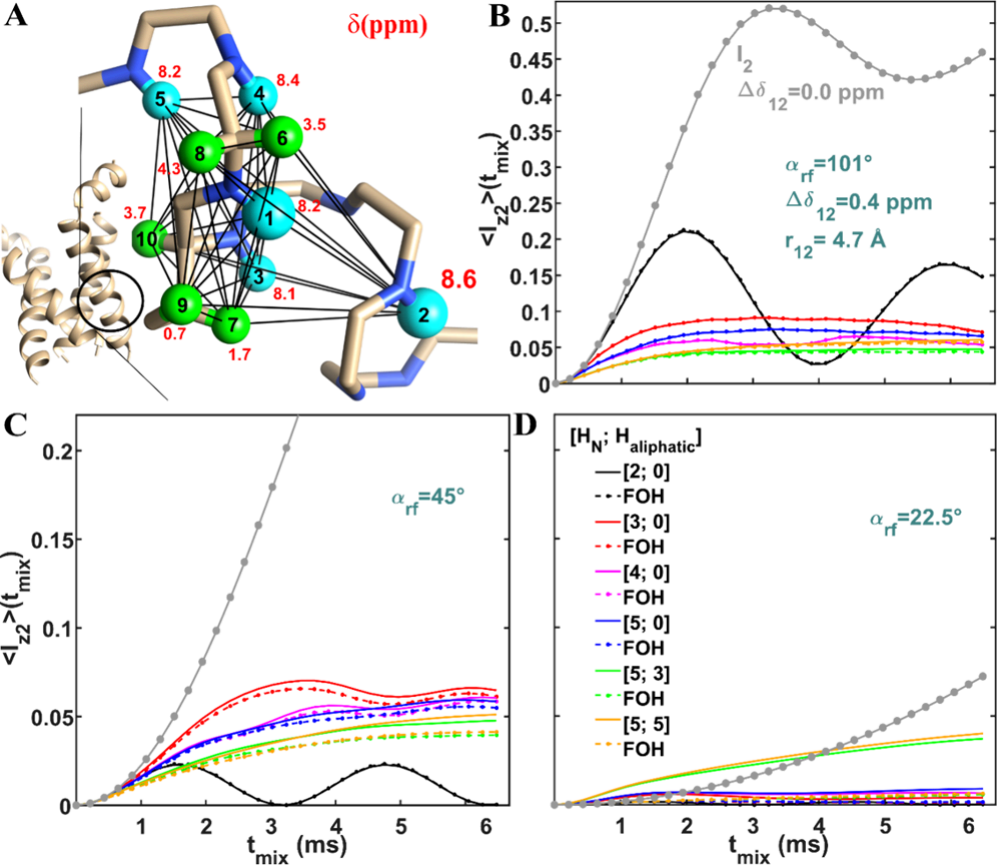
Simulations as in Figure 4, except that the offset of spin 2 was set to 8.6 ppm.

While in this specific case, the improvement in transfer efficiency is observed only for 22.5° MODIST, Figure 3 has already shown that an improvement in transfer efficiency is also expected for 45° MODIST, especially for spin pairs with larger offset differences. Figure 5 presents simulations similar to those in Figure 4, with one difference: the isotropic chemical shift difference between the initially polarized and the measured spins was doubled from 0.2 to 0.4 ppm. In this case, for 45° MODIST (Figure 5C), the inclusion of just one additional amide spin increases the transfer efficiency from 2.2% (black solid) to 7% (red solid). For 22.5° MODIST (Figure 5D), substantial improvement in transfer efficiency is observed with the addition of multiple spins including aliphatic spins (green and orange solid), and relatively little improvement is seen with addition of three amide spins. For the ten-spin system simulations (orange solid), 45° MODIST shows slightly better performance at 5% (Figure 5C) compared to 22.5° MODIST at 4% (Figure 5D)

**Figure 6:**
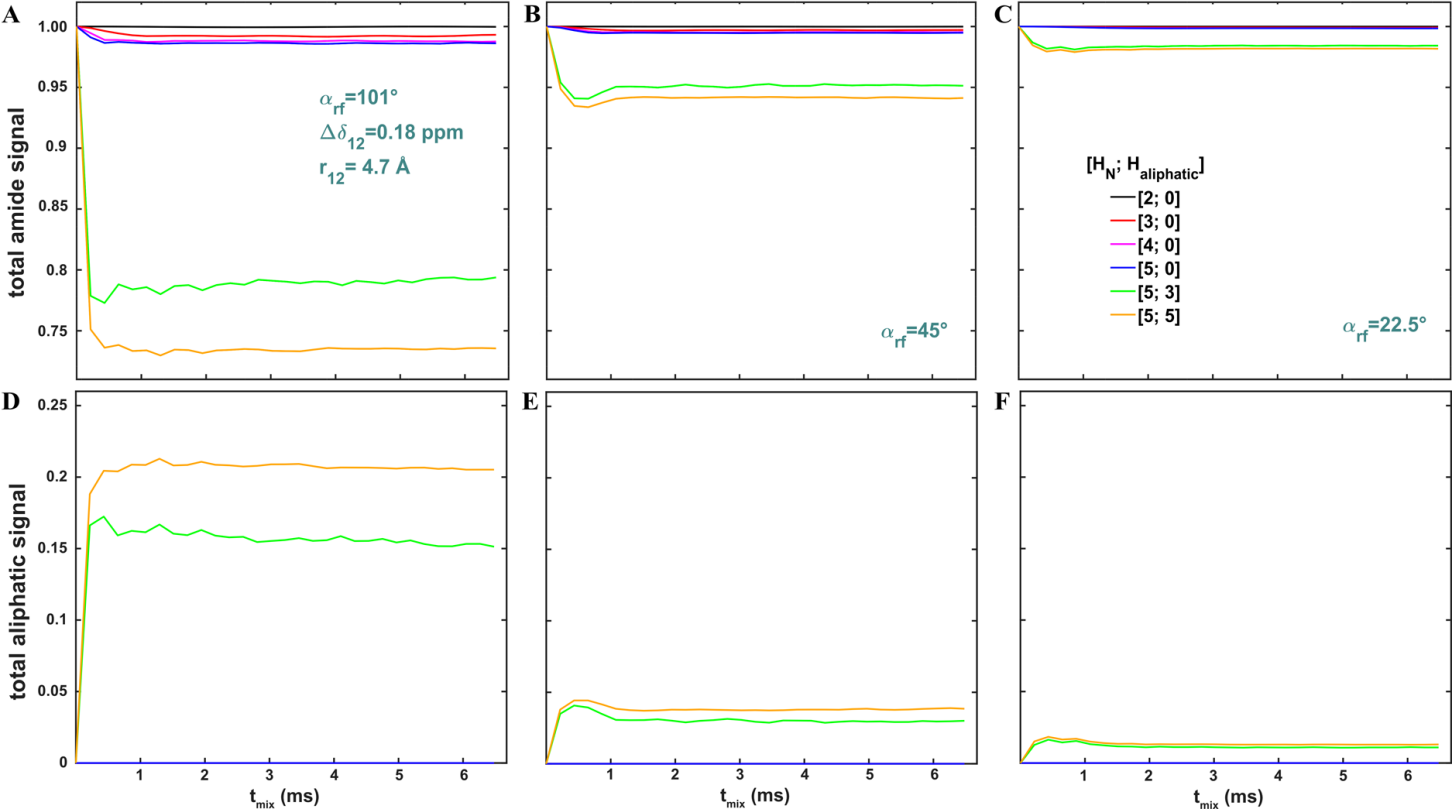
Numerical analysis of MODIST was conducted for systems comprising up to ten spins, using three different flip angles: 101° (A, D), 45° (B, E) and 22.5° (C, F). (A)-(C) The total amide signal (the signal of the spin 1 + the transferred signals to the rest of the amide spins) and (D)-(F) the total signal transferred to aliphatic spins as a function of mixing time for a series of spin systems according to the legend in (C): [amide spins; aliphatic spins]: black – [2;0]; red – [3;0]; magenta – [4;0]; blue – [5;0]; green – [5;3]; orange – [5;5]. The simulated conditions were the same as in Figure 4.

Another important parameter to consider is the total signal that remains among amide spins and the signal that is transferred to aliphatic spins (Figure 6). Both of these metrics characterize the selectivity as decreasing with increasing flip angle, but also decreasing with the number of spins in the simulation. MODIST with 45° (Figure 6B) or 22.5° (Figure 6C) flip angles preserves the total amide signal with similar efficiency up to five-spins (all amide spins, shown in black red and magenta). For the 101° flip angle (Figure 6A), signal loss of about 2% arises due to decoherence of the transferred signal as the result of powder averaging. The introduction of the aliphatic spins into the simulations affects the preservation of the total amide signal, reducing it to 0.74, 0.94, and 0.98 for 101° (orange, Figure 6A), 45° (orange, Figure 6B) and 22.5° (orange, Figure 6C), respectively. It emphasizes that for systems with high proton density, MODIST with 45° and 22.5° flip angles will be more efficient than 101° at preserving total signal in the same region. Simulations of multiple-spin systems therefore predict greater robustness for MODIST with 45° and 22.5° flip angles in selectively transferring amide signal, as compared with the 101° flip angle. Note that the 45° and 22.5° flip angles are not special and do not represent any resonance conditions.^50,103−105^Figures S2 and S3 in the SI show that any flip angle between these values provides similar transfer efficiency and effectively suppresses the undesired amide-aliphatic transfers.

According to Figure 6D-F, 101° MODIST results in undesired amide-aliphatic transfers of up to 21% for the ten-spin system with 5 aliphatic spins (orange, Figure 6D), while for the other two cases, these transfers do not exceed 4% (orange, Figure 6E-F). For MODIST with a 45° flip angle, the intensity of undesired amide-aliphatic transfers can be further reduced by placing the carrier frequency in the aliphatic region, as previously demonstrated.^85^

Note that the total signal (amide + aliphatic) does not quite reach 1 in all simulations, especially for 101° MODIST (Figures 6A and 6D), due to decoherence of the transferred signal as a result of powder averaging.

**Figure 7:**
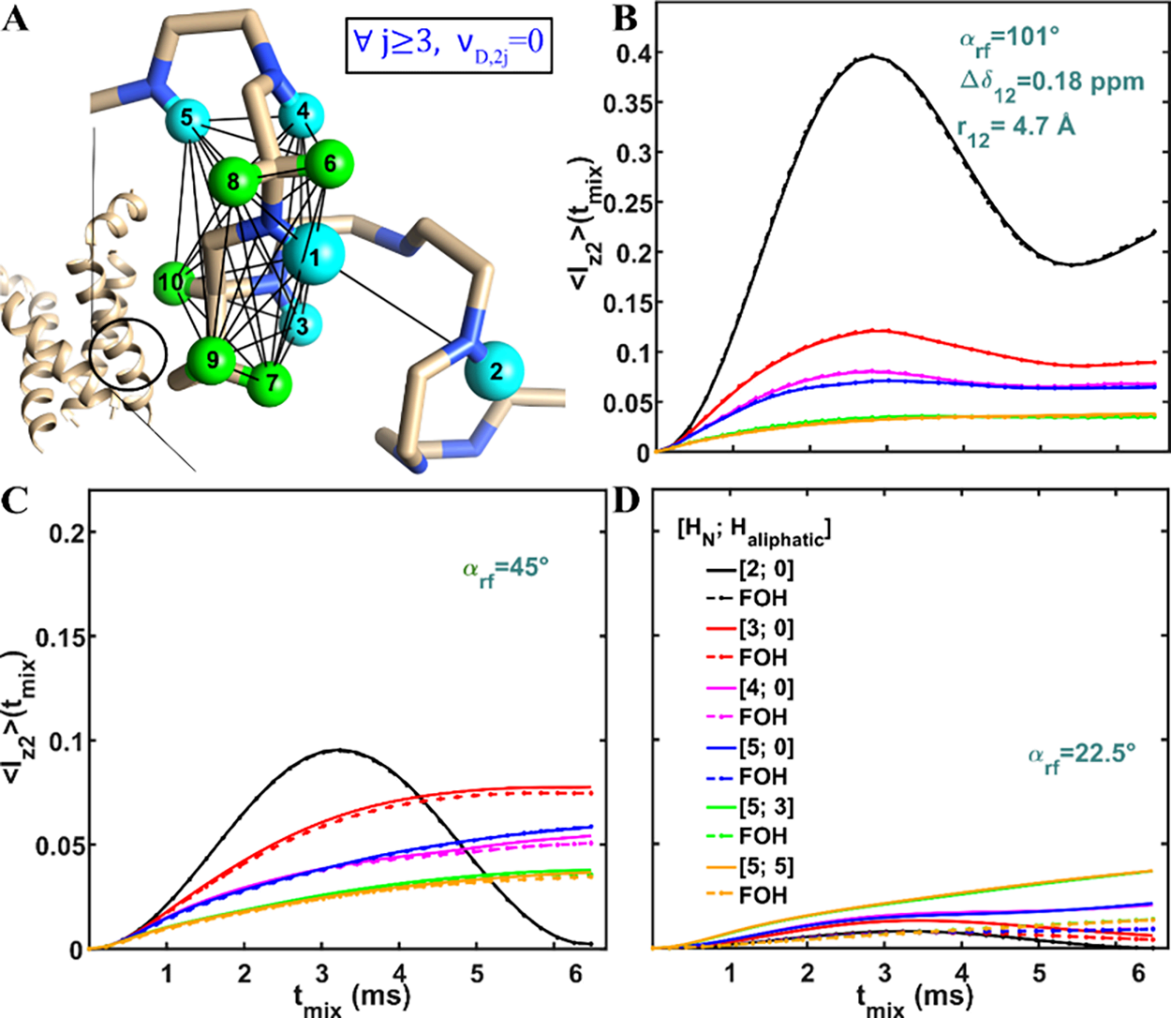
Simulations as in Figure 4, but with spin 2 only dipolar coupled with spin 1 (for *j* ≥ 3, ν_D,2j_ = 0) as depicted in (A). The structure of influenza A M2 (in A) is illustrated from the geometry found in pdb 2N70 of Andreas et al.^112^ Numerical (solid) and FOH (dashed lines with points) analysis of MODIST was conducted for systems comprising up to ten spins, using three different flip angles: 101° (B), 45° (C) and 22.5° (D).

At this point, it is unclear how much of the improvement in transfer efficiency (Figures 4 and 5) is due to FDR and how much occurs due to relayed transfer. To better isolate the FDR effect, we therefore repeated the simulation with all couplings to spin 2 turned off except the 1–2 coupling (Figure 7). It is evident that relayed transfer plays only a minor role, and the improvement in MODIST transfer efficiency for 45° (Figure 5) and 22.5° (Figures 4-5), compared with the two-spin simulations, takes place due to FDR.

**Figure 8:**
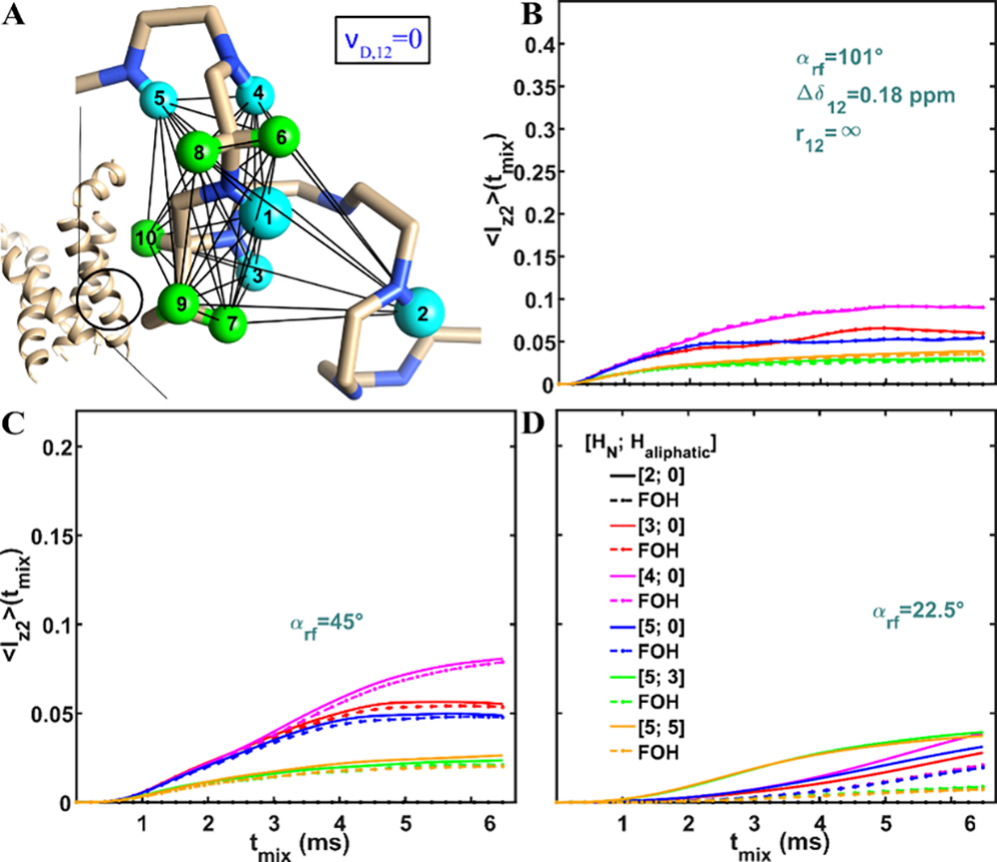
Simulations as in Figure 4, but with spin 2 isolated from spin 1 (ν_D,12_ = 0) as depicted in (A). The structure of influenza A M2 is illustrated from the geometry found in pdb 2N70 of Andreas et al.^112^ Numerical (solid) and FOH (dashed lines with points) analysis of MODIST was conducted for systems comprising up to ten spins, using three different flip angles: 101° (B), 45° (C) and 22.5° (D).

In addition to the above, the comparison of the numerical and FOH curves (Figures 4 and 5) indicates that FDR may depend on both first-order terms and higher-order terms: there is a slight disagreement between the numerical and FOH curves for the 45° flip angle (Figures 4C and 5C) and a significant discrepancy between the numerical and FOH curves for the 22.5° flip angle (Figures 4D and 5D). For first-order terms, the signal transfer can occur via direct dipolar coupling or relayed transfer.^53^ For higher-order terms, third-spin assisted recoupling^101^ may also contribute to the transfer. In Figure 8, we therefore present simulations in which we switched off the dipolar coupling between spins 1 and 2, to isolate relayed and TSAR-based transfer from direct transfer. By comparison of Figures 7 and 8, it is clear that the MODIST transfers occur via two distinct but overlapping paths. By comparison of FOH simulations with numerical simulations, it becomes clear when higher order effects become important.

First, the spin system was simulated with the second amide spin (spin 2) interacting only with the first amide spin (spin 1), isolated from the other spins, as schematically shown in the Figure 7A. Similar to Figures 4 and 5, there is full agreement between numerical (solid) and FOH (dashed with points) curves when the 101° flip angle is applied (Figure 7B). For the 45° flip angle, slight deviations occur at longer mixing times (Figure 7C). For the 22.5° flip angle (Figure 7D), similar to Figures 4D and 5D, the FOH predicts transfer efficiencies at least three times smaller than the numerical simulation (except in the two-spin system).

The next step was to simulate the spin system where the second amide spin (spin 2) was isolated from the first amide spin (spin 1) while interacting with the other spins, as schematically shown in Figure 8A. Interestingly, we observe nonzero FOH transfers for all three flip angles. For both the 101° (Figure 8B) and the 45° (Figure 8C) flip angles, the FOH curves agree with numerical curves, regardless of the spin system’s size. For the 22.5° flip angle, the FOH again predicts transfer efficiencies with smaller intensities than the numerical simulation (except in the two-spin system, where the transferred signal is zero).

According to Figures 7 and 8, for the 101° and 45° flip angles, FDR primarily depends on first-order terms, and the transfer can occur via both direct dipolar coupling (Figure 7) and relayed transfer (Figure 8). For the 22.5° flip angle, FDR mainly depends on higher-order terms, and in real samples the transfer likely occurs via a combination of mechanisms that may also involve third-spin assisted recoupling.^101^

A dependence of MODIST with a 22.5° flip angle on higher-order terms suggests that at ultrafast MAS rates, this flip angle may have less efficiency than other flip angles. Figure S4 in the SI shows the transferred signal at 110 kHz MAS. Although a 22.5° flip angle provides similar transfer efficiency compared to other flip angles, the buildup of signal is considerably slower, consistent with the presence of higher order terms in the Hamiltonian playing a role. Since relaxation effects are not included in the simulation, and since relaxation losses may be different for different flip angles, these simulations cannot be used to indicate which flip angle will be more efficient, but do suggest an increase in mixing time for 22.5° MODIST.

**Figure 9:**
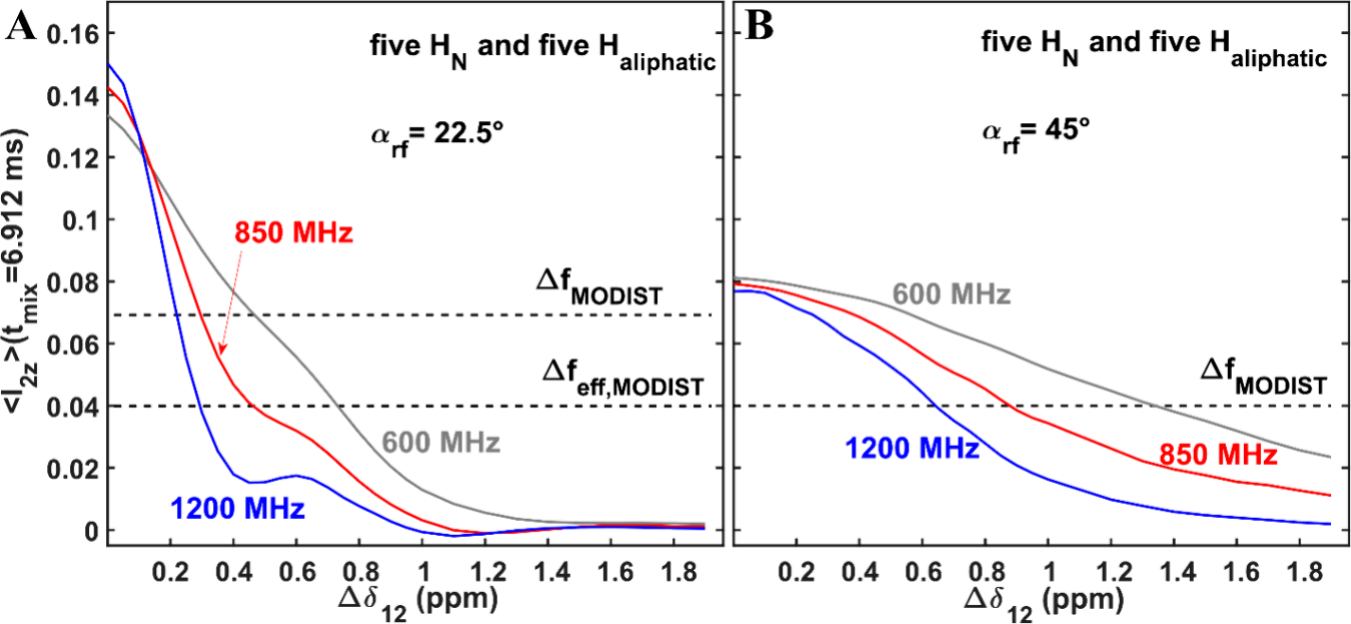
Transferred MODIST signals (from spin 1 to spin 2 for the 10-spin system in Figure 4) at 6.912 ms mixing as a function of the chemical shift difference Δδ_12_ (in ppm) for three ^1^H Larmor frequencies: 600 MHz–gray, 850 MHz–red and 1200 MHz–blue. Numerical analysis of MODIST with 22.5° (A) and 45° (B) flip angles was conducted for the ten-spin system of five amide and five aliphatic spins. The dashed line labeled Δ*f*_*MODIST*_ are drawn at ∼50% of the transferred signal with respect to the signal with Δδ_12_= 0 ppm. The dashed line labeled Δ*f*_*eff*, *MODIST*_ defines the isotropic chemical shift differences at which the transfer efficiency reaches 4%. All simulations used 55 kHz MAS and 8.54 ppm as the carrier frequency. The isotropic chemical shift of spin one was set to 8.54 ppm and the isotropic chemical shift of the spin 2 was a variable. In simulations only homonuclear dipolar interactions, isotropic chemical shifts and the applied rf-field pulses were taken into account.

So far, we have evaluated the polarization transfer between a pair of spins at a constant external magnetic field (850 MHz). It is essential to assess the dependence of the transfer efficiency on the external magnetic field. Therefore, in Figure 9, the magnetic field dependence is assessed for the 10-spin system for two flip angles: 22.5° (Figure 9A) and 45° (Figure 9B).

At Δδ_12_ = 0 ppm, MODIST with a 22.5° flip angle (Figure 9A) demonstrates nearly double the transfer efficiency compared to that with a 45° flip angle (Figure 9B). However, the 22.5° flip angle exhibits greater selectivity than the 45° flip angle. The width of selective transfer can be calculated in two ways. First, we define the isotropic chemical shift difference, denoted as Δ*f*_MODIST_, as the difference at which the transferred signal reaches 50% of the signal obtained with zero chemical shift difference. Second, we define the isotropic chemical shift difference, denoted as Δ*f*_eff, MODIST_, as the difference at which the transfer efficiency is at least 4%.

For simplicity, we have assigned identical values for Δ*f*_MODIST_ and Δ*f*_eff, MODIST_ for a 45° flip angle. Table 1 summarizes the Δ*f*_MODIST_ and Δ*f*_eff, MODIST_ values, calculated from Figure 9.

**Table 1:** Summary of Widths of the Selective Transfer at Different External Magnetic Fields and Flip Angles Obtained from 10-Spin Simulations (Figure 8)^a^

α_*rf*_	width of the selective transfer	600 MHz	850 MHz	1200 MHz
45°	Δ*f*_eff, MODIST_ = Δ*f*_MODIST_ (in ppm/in Hz)	1.35/810	0.9/765	0.67/804
22.5°	Δ*f*_MODIST_ (in ppm/in Hz)	0.45/270	0.3/255	0.2/260
	Δ*f*_eff, MODIST_ (in ppm/in Hz)	0.72/432	0.48/408	0.3/360

a Δ*f*_MODIST_ defines the isotropic chemical shift differences at which the transferred signal reaches 50% of the signal obtained at zero chemical shift difference. Δ*f*_eff, MODIST_ defines the isotropic chemical shift differences at which the transfer efficiency reaches 4%.

The effective widths of the selective transfer for the 22.5° flip angle with respect to the 45° flip angle are in an approximate ratio of 1:3 and 1:2 for Δ*f*_MODIST_ and Δ*f*_eff, MODIST_, respectively. Based on this, we can determine the conditions under which each flip angle (either 22.5° or 45°) can offer a higher transfer efficiency compared to the other.

For spin pairs with small isotropic chemical shift differences, a 22.5° flip angle will yield higher transfer efficiency than a 45° flip angle. However, regarding proton amide–amide correlations, this improvement will be noticeable primarily under moderate external magnetic fields (up to 850 MHz). In high external magnetic fields, such as 1200 MHz, a 45° flip angle will result in significantly higher transfer efficiency.

Conversely, for excitation of proton aliphatic-aliphatic correlations among similar chemical moieties, such as Ha-Ha or H_Met_-H_Met_, a 22.5° flip angle will consistently deliver significantly enhanced transfer efficiency, regardless of the field strength. This behavior stems from small differences in chemical shifts between protons of the same chemical group and the high density of strongly bonded dipolar coupled-spins. These assertions are experimentally validated in the following section.

## Results

The experimental dependence of the transfer efficiency of MODIST on flip angle values is demonstrated using uniformly ^13^C and ^15^N labeled samples of Influenza A M2 wild type (WT) and the S31N variant. Figure S5 in the SI compares 1D and 2D (H)N(H)H experiments of WT M2 with different flip angles and mixing times. The results in Figure S5 confirm the conclusions derived from simulations, namely that a large flip angle of 101° results in a significant loss of total amide signal, rendering MODIST inefficient for selective excitation of proton–proton correlations.

**Figure 10:**
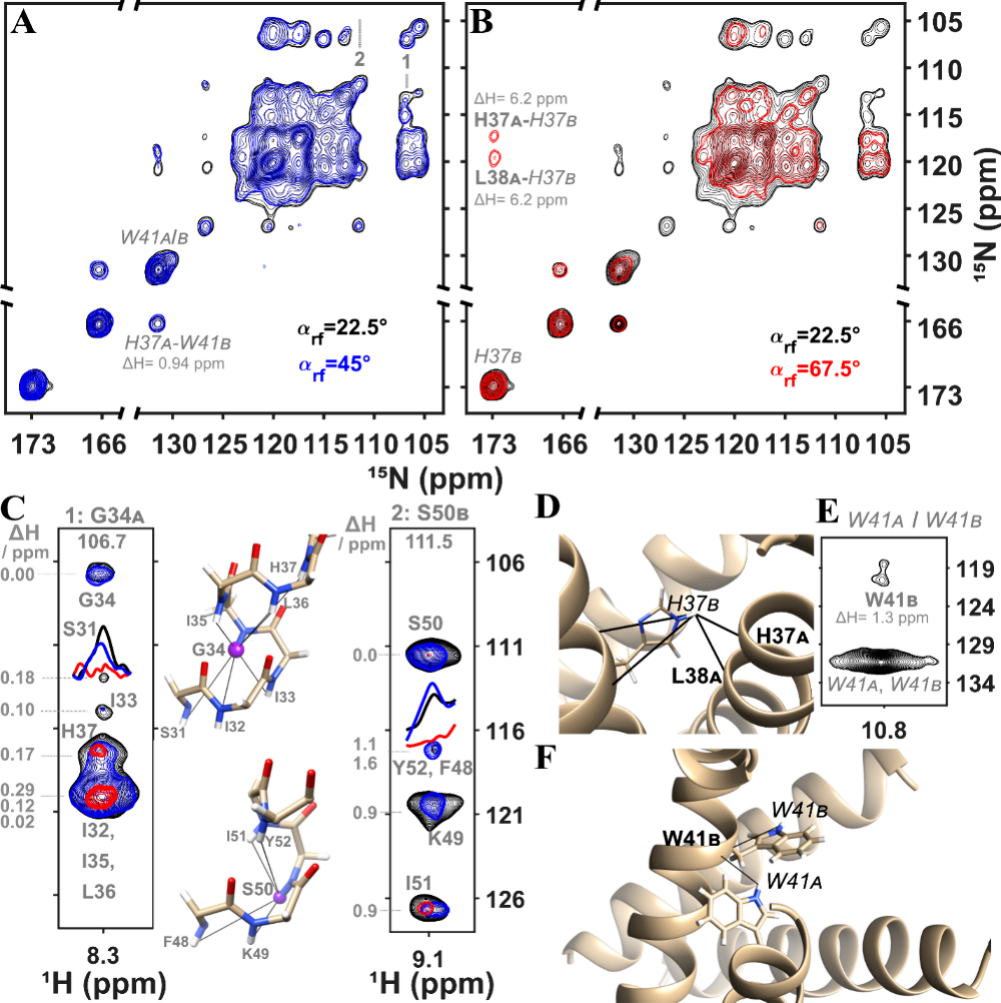
3D (H)N(H)(H)NH^MODIST^ experiments (4.608 ms mixing) were conducted with different flip angles: 67.5° - red; 45° - blue and 22.5° - black for the WT M2 sample. (A) – (B) represent ^15^N–^15^N projections. (C) and (E) show three strips extracted from the 3D spectra at the nitrogen frequencies of G34_A_ and S50_B_ (C) and overlapped W41_A_/W41_B_ (E). The inset in (C) and panels (D) and (F) display schematic illustrations of the detected contacts. The labels of aromatic spins (exchangeable protons of H and W) are *italicized*, while the amide spins are **bolded**. The structure of influenza A M2 is illustrated from the geometry found in pdb 2N70 of Andreas et al.^112^ Data were recorded at an 600 MHz spectrometer with 55.555 kHz MAS. The proton carrier frequency was set to 3 ppm. Additional experimental details are provided in the SI (Figures S9B and S14).

Figure 10 presents data obtained from 3D (H)N(H)(H)NH experiments. Figure 10A-B compares ^15^N–^15^N projections using three different flip angles: 67.5° (red), 45° (blue) and 22.5° (black). A 101° flip angle was omitted due to the substantial loss of total amide signal (Figure S5A in the SI). Additionally, Figure 10C-E displays three selected strips from the 3D experiments.

Overall, MODIST with both 45° and 22.5° flip angles demonstrates high efficiency for exciting proton amide–amide correlations, as shown previously for 45° MODIST.^85^ However, under a moderate external magnetic field of 600 MHz and for spin pairs with small offset differences, MODIST with a 22.5° flip angle (black) slightly outperforms MODIST with a 45° flip angle (blue) and shows better preservation of the total amide signal, which is also consistent with simulations (Figures 4 and 6). MODIST with a 67.5° flip angle (red) remains inefficient for exciting amide–amide correlations. This is evident from the projections in Figure 10A, B as well as the selected strips in Figure 10C-E.

Influenza A M2 is a tetrameric protein^113^ composed of two copies of two symmetry-unrelated chains, chain A and chain B.^114^ Due to the helical geometry, most cross-peaks correspond to correlations between neighboring intrachain amide protons. Both 45° and 22.5° flip angles allow the detection of correlations within a single helical turn, for instance, between G34_A_ and its neighboring residues, such as S31_A_ and H37_A_ (Figure 10C, left strip), and between S50_B_ and its neighboring residues F48_B_ and Y52_A_ (Figure 10C, right strip). While for G34_A_, the offset difference from its neighbors is around 0.2 ppm, for S50_B_, the difference is around 1 ppm. Offset differences are shown at the left of the strips in Figure 10C.

Certain interchain correlations are also observed in these experiments. The contact at a 0.94 ppm chemical shift difference between aromatic protons (H37_A_-W41_B_) is observed with all three flip angles (Figures 10A-B). An additional ambiguous contact in Figure 10E between an aromatic proton on W41_A/B_ and a backbone proton on W41_B_ (Figure 10E) is only observed with a 22.5° flip angle (illustrated schematically in Figure 10F).

Meanwhile, some contacts between aromatic and backbone amide protons with about 6.2 ppm offset difference are observed only with the less selective 67.5° flip angle (red, Figure 10B): H37_A_ (backbone) – H37_B_ (aromatic), and L38_A_ (backbone) – H37_B_ (aromatic), schematically in Figure 10D.

It is worth noting that the detection of these aromatic-backbone contacts in proton–proton mixing experiments with broad-band recoupling (RFDR)^47^ was previously accomplished for S31N M2^112^ with a perdeuterated protein. This resulted in transfer predominantly among amides due to the labeling scheme, rather than the selectivity of the recoupling.

Figure S6 further compares the performance of 22.5° (black) and 45° (blue) MODIST at an 850 MHz spectrometer, showing higher transfer efficiency for the 22.5° flip angle (black).

The performance of MODIST with various flip angles was also assessed for exciting proton aliphatic-aliphatic correlations. Figure S7 in the SI compares 2D (H)C(H)(H)C spectra using three different flip angles, recorded at two spectrometers: at 600 MHz (Figure S7A) and at 1200 MHz (Figure S7B). In both instances, the 22.5° flip angle exhibited higher transfer efficiency compared to the other two (33.75° and 45°). This indicates that for aliphatic-aliphatic correlations, a more selective MODIST is more efficient.

**Figure 11:**
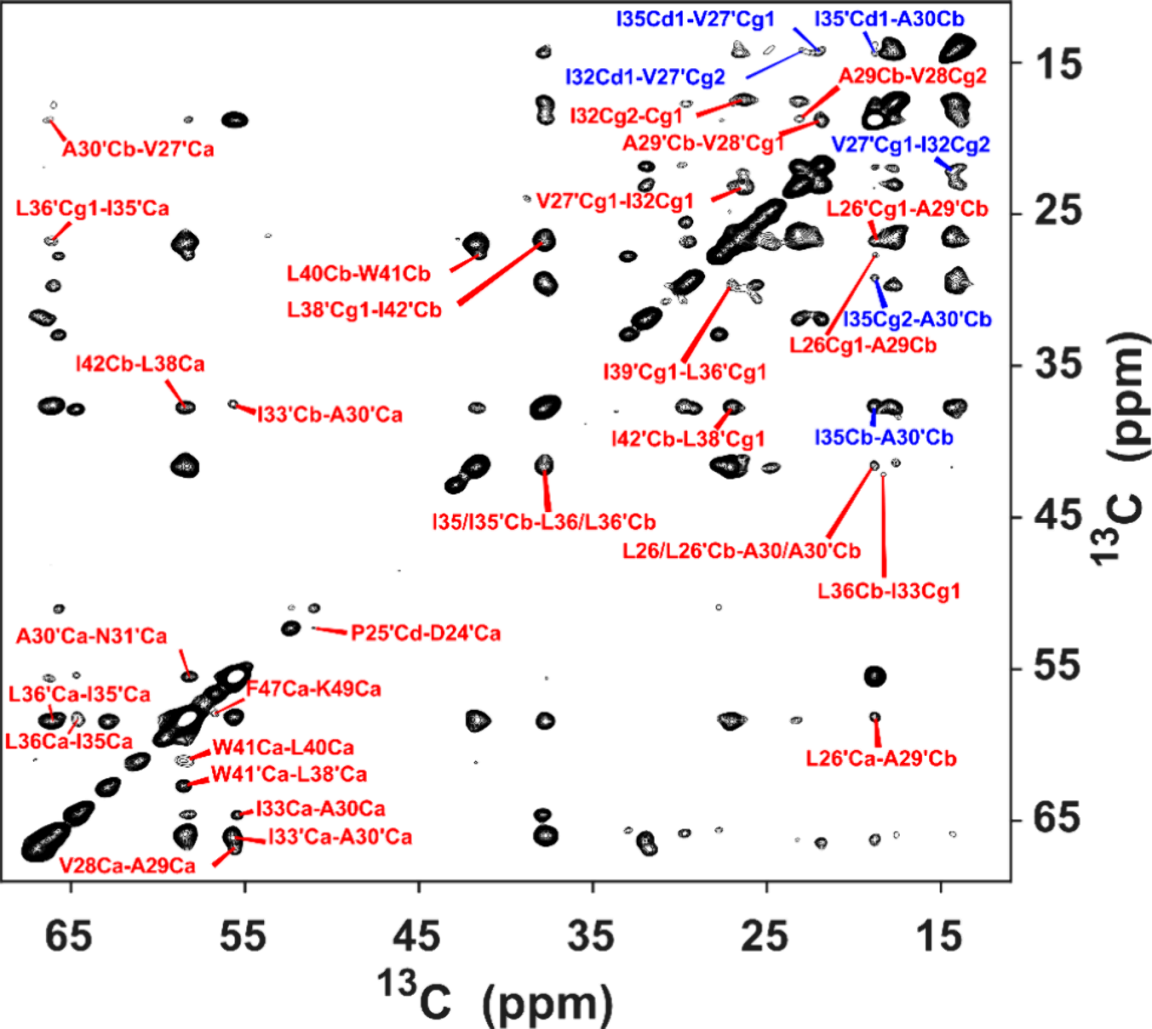
^13^C–^13^C projection from a 3D (H)C(H)(H)CH spectrum of S31N M2 showing intrahelical (red) and interhelical (blue) contacts. The proton–proton transfer was implemented with 5.76 ms MODIST mixing with a 22.5° flip angle. Data were recorded at a 1200 MHz spectrometer with 55.555 kHz MAS. The proton carrier frequency was set to 1 ppm. Additional experimental details are provided in the SI (Figures S11 and S14).

Figure 11 demonstrates the ^13^C–^13^C projection from the 3D (H)C(H)(H)CH experiment using a 22.5° flip angle for MODIST, recorded at a 1200 MHz spectrometer. This spectrum displays 34 cross-peaks in the 3D experiment, which were absent in the 2D (H)CC RFDR spectrum^47^ (Figure S8 in the SI). While most of the labeled peaks represent inter-residue proton aliphatic-aliphatic correlations within the same chain (labeled in red), some are best explained by interchain correlations, given the known tetrameric structure (labeled in blue, totaling six peaks). Many of these inter-chain contacts were previously identified in proton–proton mixing experiments with broad-band recoupling (RFDR)^47^ using a methyl-labeled S31N M2 sample (Figure 10 in ref.^112^).

## Conclusions

We used average Hamiltonian theory and numerical simulations to analyze a selective homonuclear dipolar recoupled sequence, MODIST, and identified improved experimental conditions for both amide as well as aliphatic transfer. We explored the contribution of first-order and higher order effects to the spin transfer characteristics, revealing the extent to which higher order effects and third spin effects become important depending on the spin system. Our evaluation focused on distant spin pairs (weakly dipolar-coupled spins), using the FOH solution and numerical simulations for multi-spin systems across a range of flip angle values and dipolar coupling strengths of additional dipolar-coupled spins. Specifically, we selected three flip angle values to sample a range of dipolar and isotropic chemical shift scaling factors: 101°, 45° and 22.5°. For two-spin systems, only the largest flip angle appears to be efficient, as the other two resulted in excessively narrow widths of the selective transfer, Δ*f*_*MODIST*_. However, in multispin systems, the other two flip angles (45° and 22.5°) were more efficient. Moreover, any value between these flip angles provided similar transfer efficiency and effectively suppressed the undesired amide-aliphatic transfers.

Based on multi-spin simulations and experimental measurements on a helical membrane protein, we identified the experimental conditions maximizing the transfer efficiency for each of the two flip angles: 45° (ν_*rf*_ = 0.5ν_*R*_) – for amide–amide correlations and 22.5° (ν_*rf*_ = 0.25ν_*R*_) – for aliphatic-aliphatic correlations. This optimization facilitated detection of correlations that were previously observed from perdeuterated or specifically labeled M2 samples.

A substantial difference in MODIST behavior between two-spin and multiple-spin systems was observed. We refer to this effect as ‘facilitated dipolar recoupling’ (FDR), where the addition of strong dipole couplings to the two-spin system enhances the transfer. Distinct from TSAR, the effect of the added spins is observed even when the strong couplings affect only one of the original two spins. From the comparison of the numerical and FOH curves, this influence was identified as a first-order FDR effect for 45° MODIST. For 22.5° MODIST, a first-order FDR effect only partially explains the improvement in polarization transfer. For this angle, higher-order terms that contribute to FDR, as well as third spin-assisted recoupling, should be considered.

## Supporting Information Available

Theoretical solution and numerical simulations of MODIST; additional experimental data using MODIST and RFDR sequences for dipolar recoupling; and the experimental parameters (PDF)
