# Supplementary material for: Analysis of the MODIST Sequence for Selective Proton–Proton Recoupling
Source: J Phys Chem A. 2024 Dec 23;129(1):317–29. doi: 10.1021/acs.jpca.4c05102 (PMC11726629; doi:10.1021/acs.jpca.4c05102)
Supplement: Supplementary file 1 — jp4c05102_si_001.pdf [file jp4c05102_si_001.pdf]

Analysis of the MODIST Sequence for Selective Proton-Proton Recoupling

Authors: *Evgeny Nimerovsky*, Marianna Stampolaki, Abel Cherian Varkey, Stefan Becker & Loren B. Andreas**

Affiliations:

Department of NMR based Structural Biology, Max Planck Institute for Multidisciplinary Sciences, Am Faßberg 11, Göttingen, Germany

*Corresponding authors: land@mpinat.mpg.de ORCID: 0000-0003-3216-9065 and evni@mpinat.mpg.de ORCID: 0000-0003-3002-0718.

Contents

Contents 2

Theoretical solution 2

Simulations and Experiments 12

Experimental Methods 23

Simulations 23

Sample Preparation 24

Solid state NMR spectroscopy 24

BRUKER PULSE PROGRAMS 32

3D (H)C(H)(H)CH 32

REFERENCE 37

The Supplementary Information consists of three sections. The first section, 'Theoretical Solution' details how the theoretical solution was obtained. In the second section, 'Simulations and Experiments', we show additional simulated and experimental data. In the last section, 'Experimental Methods', we provide the experimental details.

Theoretical solution

The MODIST (Modest Offset Difference Internuclear Selective Transfer) recoupling element[1] (Figure 1A in the main text) consists of sixteen pulses, with the following phase cycling: $y\bar{y}\bar{x}x x\bar{x}x\bar{y}y\bar{y}yx\bar{x}x\bar{x}y\bar{y}$. The length of each pulse is $0.25T_R$ and the flip angle, α_{rf}, is $0.5\pi\nu_{rf}T_R$. T_R $(1/\nu_R)$ and ν_{rf} represent the rotor period and the nutation frequency due to the applied rf-field, respectively. The total length of the recoupling element is $4T_R$ and can be extended in length by repetition.

For obtaining the first-order (two spin) approximate solution we apply the following main steps:

- We transform the total Hamiltonian into the tilted rf-field frame[2]

- We calculate the first order Hamiltonian.[3]

The transferred signal for the I_2 spin system with the initial and final operators, I_{1z} and I_{2z}, is described with the following equation:

$$\langle I_{2z}\rangle(t_{mix} = N4T_R) = \int d\Omega\, Tr\{I_{2z}U_{tot}(t_{mix})I_{1z}U_{tot}^{-1}(t_{mix})\}, \qquad \text{Eqn. (S1)}$$

where $d\Omega = (8\pi^2)^{-1}d\alpha d(cos\beta)d\gamma$ and the integration is performed over powder Euler angles, (α, β, γ).[2] The total propagator, $U_{tot}(t_{mix})$, consists of N repetitions of the product of sixteen propagators:

$$U_{tot}(t_{mix}) = \left(\prod_{k=16}^{1} U_k\right)^N = \left(\prod_{k=16}^{1} \hat{T}exp\left\{-i\int_{t_{k-1}}^{t_k} dt\, H_{tot,k}(t)\right\}\right)^N. \qquad \text{Eqn. (S2)}$$

where $\hat{T}$ is a Dyson operator and $H_{tot,k}$ is the total Hamiltonian of the kth-propagator:

$$H_{tot,k}(t) = H_D(t) + H_\sigma(t) + H_{rf,k}(t). \qquad \text{Eqn. (S3)}$$

$H_D(t)$ is a dipolar Hamiltonian, which is defined as follows:

$$H_D(t) = \omega_D(t)[2I_{1z}I_{2z} - 0.5(I_1^+I_2^- + I_1^-I_2^+)], \qquad \text{Eqn. (S5)}$$

where $\omega_D(t)$ is the dipolar time dependent function:[2,4] $\omega_D(t) = \sum_{n=1}^{2} C_n cos(n\omega_R t + n\gamma) = \omega_D\left(\sqrt{2}sin(2\beta)cos(\omega_R t + \gamma) - sin^2(\beta)cos(2\omega_R t + 2\gamma)\right)$ and $\omega_D = \pi\nu_{D,IS} = -\frac{\mu_0}{8\pi^2}\frac{\hbar\gamma_I\gamma_S}{r_{IS}^3}$.

$H_\sigma(t)$ is an isotropic chemical shift Hamiltonian, which is defined as follows:

$$H_\sigma(t) = \omega_1 I_{1z} + \omega_2 I_{2z}, \qquad \text{Eqn. (S5)}$$

where ω_1 and ω_2 are the chemical shift offsets.

$H_{rf,k}(t)$ is an rf-field Hamiltonian, which is defined as follows:

$$H_{rf,k}(t) = \omega_{rf}\left[(I_{1x} + I_{2x})cos\varphi_k + (I_{1y} + I_{2y})sin\varphi_k\right], \qquad \text{Eqn. (S6)}$$

where ω_{rf} is the applied rf-field strength and φ_k is the phase of the kth-pulse:

k	1	2	3	4	5	6	7	8	9	10	11	12	13	14	15	16

φ_k	0.5π	1.5π	π	0	π	0	1.5π	0.5π	1.5π	0.5π	0	π	0	π	0.5π	1.5π

Table S1 The phases of sixteen pulses of the MODIST recoupling element.

The transformation of the total Hamiltonian of the kth-propagator (Eqn. (S3)) into the tilted rf-field frame[2] is performed with the next equation:

$$H_{tot,k}^{rf} = U_{rf,k}^{-1} H_{tot,k}(t) U_{rf,k} - H_{rf,k}(t) = U_{rf,k}^{-1}[H_D(t) + H_\sigma(t)]U_{rf,k}, \qquad \text{Eqn. (S7)}$$

where $U_{rf,k}^{-1}$ is a rotation operator. It is defined as follows:

$$U_{rf,k}^{-1}(t) = \left[\prod_{l=1}^{k-1} e^{-i\varphi_l(I_{z1}+I_{z2})} e^{i\alpha(I_{x1}+I_{x2})} e^{i\varphi_l(I_{z1}+I_{z2})}\right] \times \qquad \text{Eqn. (S8)}$$

$$e^{-i\varphi_k(I_{z1}+I_{z2})} e^{i\left(\omega_{rf}t-\alpha(k-1)\right)(I_{x1}+I_{x2})} e^{i\varphi_k(I_{z1}+I_{z2})},$$

where $\alpha = \alpha_{rf} = 0.5\pi\nu_{rf}T_R$.

To simplify Eqn.(S2), we can apply average Hamiltonian theory[3], considering the first order Hamiltonian (FOH) terms:

$$U_{tot}^{rf}(t_{mix}) = \left(\prod_{k=16}^{1} \hat{T} exp\left\{-i \int_{t_{k-1}}^{t_k} dt\, H_{tot,k}^{rf}(t)\right\}\right)^N \approx exp\left\{-iN\left[4T_R H_{ave}^{(1)}\right]\right\}, \qquad \text{Eqn. (S9)}$$

where $U_{tot}^{rf}(t_{mix})$ is the total propagator in the tilted rf-field frame and $4T_R H_{ave}^{(1)}$ is:

$$4T_R H_{ave}^{(1)} = \sum_{k=1}^{16} \int_{(k-1)0.25T_R}^{k0.25T_R} dt\, H_{tot,k}^{rf}, \qquad \text{Eqn. (S10)}$$

Eq. (S8) can be rewritten by considering that the MODIST recoupling element comprises jump-return pulse pairs:[5,6] each consisting of two pulses, where the phase of the subsequent pulse is shifted by 180° compared to the first. For each odd *k*, the product of the *k-1* terms in the square brackets in Eqn. (S8) equals an identity matrix:

$$\left[\prod_{l=1}^{k-1} e^{-i\varphi_l(I_{z1}+I_{z2})} e^{i\alpha(I_{x1}+I_{x2})} e^{i\varphi_l(I_{z1}+I_{z2})}\right] = 1. \text{ For odd values of l.} \qquad \text{Eqn. (S11)}$$

Therefore, the modified Eq. (S8) when k is an odd number is:

$$U^{-1}_{rf,k} = e^{-i\varphi_k(I_{z1}+I_{z2})} e^{i(\omega_{rf}t-\alpha(k-1))(I_{x1}+I_{x2})} e^{i\varphi_k(I_{z1}+I_{z2})}. \quad \text{Eqn. (S12)}$$

For when k is an even number, the Eqn. (S8) can be rewritten as follows:

$$U^{-1}_{rf,k} = e^{-i\varphi_{k-1}(I_{z1}+I_{z2})} e^{i\alpha(I_{x1}+I_{x2})} e^{i\varphi_{k-1}(I_{z1}+I_{z2})} e^{-i\varphi_k(I_{z1}+I_{z2})} \times e^{i(\omega_{rf}t-\alpha(k-1))(I_{x1}+I_{x2})} e^{i\varphi_k(I_{z1}+I_{z2})} = e^{-i\varphi_k(I_{z1}+I_{z2})} e^{-i(\varphi_{k-1}-\varphi_k)(I_{z1}+I_{z2})} \times e^{i\alpha(I_{x1}+I_{x2})} e^{i(\varphi_{k-1}-\varphi_k)(I_{z1}+I_{z2})} e^{i(\omega_{rf}t-\alpha(k-1))(I_{x1}+I_{x2})} e^{i\varphi_k(I_{z1}+I_{z2})}. \quad \text{Eqn. (S13)}$$

The difference, $\varphi_{k-1} - \varphi_k$, in Eqn. (S13) equals to:

k	2	4	6	8	10	12	14	16
$\varphi_{k-1} - \varphi_k$	-π	π	π	π	π	-π	-π	-π

Table S2 The phase difference, $\varphi_{k-1} - \varphi_k$, for even values of k up to 16. φ_k values are shown in Table S1.

According to Table S2 the following product (the central part of Eqn. (S13)) can be rewritten as:

$$e^{-i(\varphi_{k-1}-\varphi_k)(I_{z1}+I_{z2})} e^{i\alpha(I_{x1}+I_{x2})} e^{i(\varphi_{k-1}-\varphi_k)(I_{z1}+I_{z2})} = e^{-i\alpha(I_{x1}+I_{x2})}. \quad \text{Eqn. (S14)}$$

Substituting Eqn. (S14) into Eqn. (S13), the modified Eqn. (S13) is:

$$U^{-1}_{rf,k} = e^{-i\varphi_k(I_{z1}+I_{z2})} e^{-i\alpha(I_{x1}+I_{x2})} e^{i(\omega_{rf}t-\alpha(k-1))(I_{x1}+I_{x2})} e^{i\varphi_k(I_{z1}+I_{z2})} = e^{-i\varphi_k(I_{z1}+I_{z2})} e^{i(\omega_{rf}t-\alpha k)(I_{x1}+I_{x2})} e^{i\varphi_k(I_{z1}+I_{z2})}. \quad \text{Eqn. (S15)}$$

For any k, either odd or even, Eqns. (S12) and (S15) can be written as follows:

$$U^{-1}_{rf,k} = e^{-i\varphi_k(I_{z1}+I_{z2})} e^{i(\omega_{rf}t-\phi_k)(I_{x1}+I_{x2})} e^{i\varphi_k(I_{z1}+I_{z2})}, \quad \text{Eqn. (S16)}$$

where φ_k and ϕ_k values for sixteen blocks are defined as follows:

k	1	2	3	4	5	6	7	8	9	10	11	12	13	14	15	16
φ_k	$\frac{\pi}{2}$	$\frac{3\pi}{2}$	π	0	π	0	$\frac{3\pi}{2}$	$\frac{\pi}{2}$	$\frac{3\pi}{2}$	$\frac{\pi}{2}$	0	π	0	π	$\frac{\pi}{2}$	$\frac{3\pi}{2}$
ϕ_k	0	2α	2α	4α	4α	6α	6α	8α	8α	10α	10α	12α	12α	14α	14α	16α

Table S3 The summary of φ_k and ϕ_k phases.

To simplify the calculations of the transformation of the total Hamiltonian into the tilted rf-field frame (Eqn. (S7)) and the FOH (Eqn. (S10)), we consider the dipolar and isotropic chemical shift interactions separately and then summarize the results.

The dipolar Hamiltonian in the titled frame is:

$$H_{D,k}^{rf} = U_{rf,k}^{-1} H_D(t) U_{rf,k} = \omega_D(t) U_{rf,k}^{-1}[2I_{1z}I_{2z} - 0.5(I_1^+ I_2^- + I_1^- I_2^+)]U_{rf,k}. \qquad \text{Eqn. (S17)}$$

First, we evaluate the secular part of the dipolar Hamiltonian in Eqn. (S17), using Eqn. (S16) for $U_{rf,k}^{-1}$:

$$U_{rf,k}^{-1} I_{z1} I_{z2} U_{rf,k} = [I_{1z}\cos(\omega_{rf}t - \phi_k) + e^{-i\varphi_k I_{1z}} I_{1y} e^{i\varphi_k I_{1z}} \sin(\omega_{rf}t - \phi_k)] \cdot [I_{2z}\cos(\omega_{rf}t - \phi_k) + e^{-i\varphi_k I_{2z}} I_{2y} e^{i\varphi_k I_{2z}} \sin(\omega_{rf}t - \phi_k)] = [I_{1z}I_{2z}\cos^2(\omega_{rf}t - \phi_k) + e^{-i\varphi_k(I_{1z}+I_{2z})} I_{1y}I_{2y} e^{i\varphi_k(I_{1z}+I_{2z})}\sin^2(\omega_{rf}t - \phi_k) + (I_{1z}e^{-i\varphi_k I_{2z}} I_{2y} e^{i\varphi_k I_{2z}} + e^{-i\varphi_k I_{1z}} I_{1y} e^{i\varphi_k I_{1z}} I_{2z}) 0.5\sin(2\omega_{rf}t - 2\phi_k)]. \qquad \text{Eqn. (S18)}$$

The flip-flop part of the dipolar Hamiltonian in Eqn. (S17) in the titled rf-field frame is:

$$U_{rf,k}^{-1} 0.5[I_1^+ I_2^- + I_1^- I_2^+]U_{rf,k} = [I_{1z}I_{2z}\sin^2(\omega_{rf}t - \phi_k) + e^{-i\varphi_k(I_{1z}+I_{2z})} I_{1x}I_{2x} e^{i\varphi_k(I_{1z}+I_{2z})} + e^{-i\varphi_k(I_{1z}+I_{2z})} I_{1y}I_{2y} e^{i\varphi_k(I_{1z}+I_{2z})}\cos^2(\omega_{rf}t - \phi_k) - (I_{1z}e^{-i\varphi_k I_{2z}} I_{2y} e^{i\varphi_k I_{2z}} + e^{-i\varphi_k I_{1z}} I_{1y} e^{i\varphi_k I_{1z}} I_{2z}) 0.5\sin(2\omega_{rf}t - 2\phi_k). \qquad \text{Eqn. (S19)}$$

Substituting Eqns. (S18) and (S19) into Eqn. (S17), the modified Eqn. (S17) is:

$$H_{D,k}^{rf} = 2\omega_D(t)\{I_{1z}I_{2z}\cos^2(\omega_{rf}t - \phi_k) + e^{-i\varphi_k(I_{1z}+I_{2z})} I_{1y}I_{2y} e^{i\varphi_k(I_{1z}+I_{2z})}\sin^2(\omega_{rf}t - \phi_k) + (I_{1z}e^{-i\varphi_k I_{2z}} I_{2y} e^{i\varphi_k I_{2z}} + e^{-i\varphi_k I_{1z}} I_{1y} e^{i\varphi_k I_{1z}} I_{2z}) 0.5\sin(2\omega_{rf}t - 2\phi_k)\} - \omega_D(t)\{I_{1z}I_{2z}\sin^2(\omega_{rf}t - \phi_k) + e^{-i\varphi_k(I_{1z}+I_{2z})} I_{1x}I_{2x} e^{i\varphi_k(I_{1z}+I_{2z})} + e^{-i\varphi_k(I_{1z}+I_{2z})} I_{1y}I_{2y} e^{i\varphi_k(I_{1z}+I_{2z})}\cos^2(\omega_{rf}t - \phi_k) - (I_{1z}e^{-i\varphi_k I_{2z}} I_{2y} e^{i\varphi_k I_{2z}} + e^{-i\varphi_k I_{1z}} I_{1y} e^{i\varphi_k I_{1z}} I_{2z}) 0.5\sin(2\omega_{rf}t - 2\phi_k)\} = \omega_D(t)\{I_{1z}I_{2z}[1 + \cos(2\omega_{rf}t - 2\phi_k) - 0.5 + 0.5\cos(2\omega_{rf}t - 2\phi_k)] + e^{-i\varphi_k(I_{1z}+I_{2z})} I_{1y}I_{2y} e^{i\varphi_k(I_{1z}+I_{2z})}[1 - \cos(2\omega_{rf}t - 2\phi_k) - 0.5 - 0.5\cos(2\omega_{rf}t - 2\phi_k)] + 1.5[I_{1z}e^{-i\varphi_k I_{2z}} I_{2y} e^{i\varphi_k I_{2z}} + e^{-i\varphi_k I_{1z}} I_{1y} e^{i\varphi_k I_{1z}} I_{2z}]\sin(2\omega_{rf}t - 2\phi_k) - e^{-i\varphi_k(I_{1z}+I_{2z})} I_{1x}I_{2x} e^{i\varphi_k(I_{1z}+I_{2z})} = 1.5\omega_D(t)\{I_{1z}I_{2z}\cos(2\omega_{rf}t - 2\phi_k) - e^{-i\varphi_k(I_{1z}+I_{2z})} I_{1y}I_{2y} e^{i\varphi_k(I_{1z}+I_{2z})}\cos(2\omega_{rf}t - 2\phi_k)\} + \boldsymbol{\omega_D(t)\{0.5 I_{1z}I_{2z} + 0.5 e^{-i\varphi_k(I_{1z}+I_{2z})} I_{1y}I_{2y} e^{i\varphi_k(I_{1z}+I_{2z})} - e^{-i\varphi_k(I_{1z}+I_{2z})} I_{1x}I_{2x} e^{i\varphi_k(I_{1z}+I_{2z})} + 1.5(I_{1z}e^{-i\varphi_k I_{2z}} I_{2y} e^{i\varphi_k I_{2z}} + e^{-i\varphi_k I_{1z}} I_{1y} e^{i\varphi_k I_{1z}} I_{2z})\sin(2\omega_{rf}t - 2\phi_k)\}} = H_{D,k;t}^{rf} + \boldsymbol{H_{D,k;c}^{rf}}. \qquad \text{Eqn. (S20)}$$

The first order Hamiltonian term is:

$$4T_R H_{ave,D}^{(1)} = \sum_{k=1}^{16} \int_{(k-1)0.25T_R}^{k0.25T_R} dt \left[H_{D,k;t}^{rf} + \boldsymbol{H_{D,k;c}^{rf}} \right]. \qquad \text{Eqn. (S21)}$$

For $\boldsymbol{H_{D,k;c}^{rf}}$, the summation of the integrated terms provides zero. Therefore, Eqn. (S21) can be written as follows:

$$4T_R H_{ave,D}^{(1)} = \sum_{k=1}^{16} \int_{(k-1)0.25T_R}^{k0.25T_R} dt\, H_{D,k;t}^{rf} = \sum_{k=1}^{16} \int_{(k-1)0.25T_R}^{k0.25T_R} dt\, 1.5\omega_D(t) \times \qquad \text{Eqn. (S22)}$$

$$\{I_{1z}I_{2z}\cos(2\omega_{rf}t - 2\phi_k) - e^{-i\varphi_k(I_{1z}+I_{2z})} I_{1y}I_{2y} e^{i\varphi_k(I_{1z}+I_{2z})} \cos(2\omega_{rf}t - 2\phi_k)\} = 1.5 I_{1z}I_{2z} \sum_{k=1}^{16} F_{D1,k}^{(1)} + 1.5 \sum_{k=1}^{16} e^{-i\varphi_k(I_{1z}+I_{2z})} I_{1y}I_{2y} e^{i\varphi_k(I_{1z}+I_{2z})} F_{D2,k}^{(2)}\,.$$

We next evaluate the integral for the first term in Eqn. (S22), $F_{D1,k}^{(1)}$:

$$F_{D1,k}^{(1)} = \int_{(k-1)0.25T_R}^{k0.25T_R} dt\, \omega_D(t)\cos(2\omega_{rf}t - 2\phi_k) =$$

$$\sum_{n=1}^{2} C_n cos(n\omega_R t + n\gamma)\cos(2\omega_{rf}t - 2\phi_k) = \sum_{n=1}^{2} 0.5C_n \int_{(k-1)0.25T_R}^{k0.25T_R} dt \left[cos\left[(n\omega_R + 2\omega_{rf})t + n\gamma - 2\phi_k\right] + cos\left[(n\omega_R - 2\omega_{rf})t + n\gamma + 2\phi_k\right]\right] = \sum_{n=1}^{2} 0.5C_n \{\frac{1}{(n\omega_R+2\omega_{rf})}[sin\left((n\omega_R + 2\omega_{rf})k0.25T_R + n\gamma - 2\phi_k\right) - sin\left((n\omega_R + 2\omega_{rf})(k-1)0.25T_R + n\gamma - 2\phi_k\right)] + \frac{1}{(n\omega_R-2\omega_{rf})}[sin\left((n\omega_R - 2\omega_{rf})k0.25T_R + n\gamma + 2\phi_k\right) - sin\left((n\omega_R - 2\omega_{rf})(k-1)0.25T_R + n\gamma + 2\phi_k\right)]\} = \qquad \text{Eqn. (S23)}$$

$$\sum_{n=1}^{2} C_n \{\frac{1}{(n\omega_R + 2\omega_{rf})} sin\left((n\omega_R + 2\omega_{rf})0.125T_R\right) \cdot cos\left((n\omega_R + 2\omega_{rf})k0.25T_R + n\gamma - 2\phi_k - (n\omega_R + 2\omega_{rf})0.125T_R\right) + \frac{1}{(n\omega_R-2\omega_{rf})} sin\left((n\omega_R - 2\omega_{rf})0.125T_R\right) cos\left((n\omega_R - 2\omega_{rf})k0.25T_R + n\gamma + 2\phi_k - (n\omega_R - 2\omega_{rf})0.125T_R\right)\} =$$

$$\sum_{n=1}^{2} C_n \{\frac{1}{(n\omega_R+2\omega_{rf})} sin(0.25n\pi + \alpha)\, cos((k-0.5)0.5\pi n + n\gamma + 2k\alpha - \alpha - 2\phi_k) + \frac{1}{(n\omega_R-2\omega_{rf})} sin(0.25n\pi - \alpha) cos((k-0.5)0.5\pi n + n\gamma - (2k\alpha - \alpha - 2\phi_k))\}.$$

We define $2k\alpha - \alpha - 2\phi_k$ as Φ_k and use ϕ_k values from Table S3. Φ_k for sixteen blocks is summarized in the Table S4.

k	1	2	3	4	5	6	7	8	9	10	11	12	13	14	15	16
ϕ_k	0	2α	2α	4α	4α	6α	6α	8α	8α	10α	10α	12α	12α	14α	14α	16α
Φ_k	α	$-\alpha$	α	$-\alpha$	α	$-\alpha$	α	$-\alpha$	α	$-\alpha$	α	$-\alpha$	α	$-\alpha$	α	$-\alpha$

Table S4 The summary of ϕ_k and Φ_k phases.

According to the Table S4, Eqn. (S23) can be rewritten as follows:

$$F_{D1,k}^{(1)} = \sum_{n=1}^{2} C_n \left\{\frac{1}{(n\omega_R+2\omega_{rf})} sin(0.25n\pi+\alpha)\, cos\left((k-0.5)0.5\pi n + n\gamma - (-1)^k\alpha\right) + \frac{1}{(n\omega_R-2\omega_{rf})} sin(0.25n\pi-\alpha) cos\left((k-0.5)0.5\pi n + n\gamma + (-1)^k\alpha\right)\right\}. \quad \text{Eqn. (S24)}$$

The summation of Eqn. (S24) over k between 1 and 16 (from Eqn. (S22)) results in:

$$1.5I_{1z}I_{2z}\sum_{k=1}^{16} F_{D1,k}^{(1)} = 1.5I_{1z}I_{2z}\omega_D \sum_{k=1}^{16}\sum_{n=1}^{2} C_n \left\{\frac{1}{(n\omega_R+2\omega_{rf})} sin(0.25n\pi+\alpha)\, cos\left((k-0.5)0.5\pi n + n\gamma - (-1)^k\alpha\right) + \frac{1}{(n\omega_R-2\omega_{rf})} sin(0.25n\pi-\alpha) cos\left((k-0.5)0.5\pi n + n\gamma + (-1)^k\alpha\right)\right\} = 6I_{1z}I_{2z}\omega_D \sum_{k=1}^{4}\sum_{n=1}^{2} C_n \left\{\frac{1}{(n\omega_R+2\omega_{rf})} sin(0.25n\pi+\alpha)\, cos\left((k-0.5)0.5\pi n + n\gamma - (-1)^k\alpha\right) + \frac{1}{(n\omega_R-2\omega_{rf})} sin(0.25n\pi-\alpha) cos\left((k-0.5)0.5\pi n + n\gamma + (-1)^k\alpha\right)\right\}. \quad \text{Eqn. (S25)}$$

Since for n=1 the summation over k terms in Eqn. (S25) provides zero contribution:

$$1.5I_{1z}I_{2z}\sum_{k=1}^{16} F_{D1,k}^{(1)} = 6I_{1z}I_{2z}C_1\omega_D \sum_{k=1}^{4}\left\{\frac{sin(0.25\pi+\alpha)}{(\omega_R+2\omega_{rf})}\, cos\left((k-0.5)0.5\pi + \gamma - (-1)^k\alpha\right) + \frac{sin(0.25\pi-\alpha)}{(\omega_R-2\omega_{rf})} cos\left((k-0.5)0.5\pi + \gamma + (-1)^k\alpha\right)\right\} = 6I_{1z}I_{2z}C_1\omega_D \frac{sin(0.25\pi+\alpha)}{(\omega_R+2\omega_{rf})}\{cos(0.25\pi+\gamma+\alpha) + cos(0.75\pi+\gamma-\alpha) + cos(1.25\pi+\gamma+\alpha) + cos(1.75\pi+\gamma-\alpha)\} + \frac{sin(0.25\pi-\alpha)}{(\omega_R-2\omega_{rf})}\{cos(0.25\pi+\gamma-\alpha) + cos(0.75\pi+\gamma+\alpha) + cos(1.25\pi+\gamma-\alpha) + cos(1.75\pi+\gamma+\alpha)\} = 0, \quad \text{Eqn. (S26)}$$

then Eqn. (S25) can be rewritten as:

$$1.5I_{1z}I_{2z}\sum_{k=1}^{16} F_{D1,k}^{(1)} = 6I_{1z}I_{2z}\omega_D C_2 \sum_{k=1}^{4}\left\{\frac{1}{(2\omega_R+2\omega_{rf})} sin(0.5\pi+\alpha)\, cos\left((k-0.5)\pi + 2\gamma - (-1)^k\alpha\right) + \frac{1}{(2\omega_R-2\omega_{rf})} sin(0.5\pi-\alpha) cos\left((k-0.5)\pi + 2\gamma + \right.\right. \quad \text{Eqn. (S27)}$$

$(-1)^k\alpha\Big)\}=6I_{1z}I_{2z}\omega_D C_2\sum_{k=1}^{2}\{\frac{1}{(\omega_R+\omega_{rf})}sin(0.5\pi+\alpha)\,cos\Big((k-0.5)\pi+2\gamma-(-1)^k\alpha\Big)+\frac{1}{(\omega_R-\omega_{rf})}sin(0.5\pi-\alpha)cos\Big((k-0.5)\pi+2\gamma+(-1)^k\alpha\Big)\}=$

$6I_{1z}I_{2z}\omega_D C_2 cos(\alpha)\{\frac{1}{(\omega_R+\omega_{rf})}\big(cos(0.5\pi+2\gamma+\alpha)+cos(1.5\pi+2\gamma-\alpha)\big)+\frac{1}{(\omega_R+\omega_{rf})}\big(cos(0.5\pi+2\gamma-\alpha)+cos(1.5\pi+2\gamma+\alpha)\big)\}=$
$-6I_{1z}I_{2z}C_2\omega_D sin(2\alpha)cos(2\gamma)\frac{2\omega_{rf}}{(\omega_R{}^2-\omega_{rf}{}^2)}.$

To calculate the second term, $F_{D2,k}^{(2)}$, in Eqn. (S22), we use φ_k from Table S3:

$$1.5\sum_{k=1}^{16}e^{-i\varphi_k(I_{1z}+I_{2z})}I_{1y}I_{2y}e^{i\varphi_k(I_{1z}+I_{2z})}F_{D2,k}^{(1)}=$$ Eqn. (S28)

$-1.5\sum_{k=1}^{16}e^{-i\varphi_k(I_{1z}+I_{2z})}I_{1y}I_{2y}e^{i\varphi_k(I_{1z}+I_{2z})}\int_{(k-1)0.25T_R}^{k0.25T_R}dt\,\omega_D(t)\cos\big(2\omega_{rf}t-2\phi_k\big)=$
$-1.5\sum_{k=1}^{16}e^{-i\varphi_k(I_{1z}+I_{2z})}I_{1y}I_{2y}e^{i\varphi_k(I_{1z}+I_{2z})}\,\omega_D\sum_{n=1}^{2}C_n\,\{\frac{1}{(n\omega_R+2\omega_{rf})}sin(0.25n\pi+\alpha)\times cos\Big((k-0.5)0.5\pi n+n\gamma-(-1)^k\alpha\Big)+\frac{1}{(n\omega_R-2\omega_{rf})}sin(0.25n\pi-\alpha)cos\Big((k-0.5)0.5\pi n+n\gamma+(-1)^k\alpha\Big)\}.$

Similar to Eqn. (S25) the terms with n=1 equal to zero. Eqn. (S28) can be simplified further if we take into account that for n=2 terms:

$$\sum_{k=1}^{16}e^{-i\varphi_k(I_{1z}+I_{2z})}I_{1y}I_{2y}e^{i\varphi_k(I_{1z}+I_{2z})}F_{D2,k}^{(1)}=4\sum_{k=1}^{4}e^{-i\varphi_k(I_{1z}+I_{2z})}I_{1y}I_{2y}e^{i\varphi_k(I_{1z}+I_{2z})}F_{D2,k}^{(1)}.$$ Eqn. (S29)

Therefore, the modified Eqn. (S28) is:

$$1.5\sum_{k=1}^{16}e^{-i\varphi_k(I_{1z}+I_{2z})}I_{1y}I_{2y}e^{i\varphi_k(I_{1z}+I_{2z})}F_{D2,k}^{(1)}$$ Eqn. (S30)

$$=-6\sum_{k=1}^{4}e^{-i\varphi_k(I_{1z}+I_{2z})}I_{1y}I_{2y}e^{i\varphi_k(I_{1z}+I_{2z})}\,\omega_D C_2\times$$

$\{\frac{1}{(2\omega_R+2\omega_{rf})}sin(0.5\pi+\alpha)cos\Big((k-0.5)\pi+2\gamma-(-1)^k\alpha\Big)+\frac{1}{(2\omega_R-2\omega_{rf})}sin(0.5\pi-\alpha)cos\Big((k-0.5)\pi+2\gamma+(-1)^k\alpha\Big)\}=3\big[I_{1x}I_{2x}+I_{1y}I_{2y}\big]cos(\alpha)\omega_D C_2[\frac{1}{(\omega_R+\omega_{rf})}\big(-2cos(2\gamma)\big)sin(\alpha)+\frac{1}{(\omega_R-\omega_{rf})}2cos(2\gamma)sin(\alpha)]=$

$3[I_{1x}I_{2x} + I_{1y}I_{2y}]sin(2\alpha)\omega_D C_2 cos(2\gamma)\frac{2\omega_{rf}}{(\omega_R{}^2-\omega_{rf}{}^2)}$.

The sum of Eqns. (S27) and (S30) gives the FOH for the dipolar interaction:

$$4T_R H^{(1)}_{ave,D} = -6I_{1z}I_{2z}C_2\omega_D sin(2\alpha)cos(2\gamma)\frac{2\omega_{rf}}{(\omega_R{}^2-\omega_{rf}{}^2)} + 3[I_{1x}I_{2x} + I_{1y}I_{2y}]sin(2\alpha)\omega_D C_2 cos(2\gamma)\frac{2\omega_{rf}}{(\omega_R{}^2-\omega_{rf}{}^2)} = \{2I_{1z}I_{2z} - I_{1x}I_{2x} - I_{1y}I_{2y}\}3sin(2\alpha)sin^2(\beta)cos(2\gamma)(\nu_D T_R)\frac{\nu_{rf}/\nu_R}{(1-(\nu_{rf}/\nu_R)^2)},$$ Eqn. (S31)

where we replace ω_D by $\omega_D = 2\pi\nu_D = \nu_D T_R/(\omega_R)$, $C_2 = -\frac{sin^2(\beta)}{2}$ and $\omega_{rf}/\omega_R = \nu_{rf}/\nu_R$.

For isotropic chemical shift interaction, the isotropic chemical shift Hamiltonian in the titled frame is:

$$H^{rf}_{\sigma,k} = U^{-1}_{rf,k}H_\sigma(t)U_{rf,k} = \omega_D(t)U^{-1}_{rf,k}[\omega_1 I_{1z} + \omega_2 I_{2z}]U_{rf,k} = \sum_{o=1}^{2} U^{-1}_{rf,k}\omega_o I_{oz}U_{rf,k} = \sum_{o=1}^{2}\omega_o[I_{oz}cos(\omega_{rf}t-\phi_k) + e^{-i\varphi_k I_{oz}}I_{oy}e^{i\varphi_k I_{oz}}sin(\omega_{rf}t-\phi_k)].$$ Eqn. (S32)

The FOH of isotropic chemical shift Hamiltonian is:

$$4T_R H^{(1)}_{ave,\sigma} = \sum_{k=1}^{16}\int_{(k-1)0.25T_R}^{k0.25T_R} dt\, H^{rf}_{\sigma,k} = \sum_{k=1}^{16}\int_{(k-1)0.25T_R}^{k0.25T_R} dt\,\{\sum_{o=1}^{2}\omega_o[I_{oz}cos(\omega_{rf}t-\phi_k) + e^{-i\varphi_k I_{oz}}I_{oy}e^{i\varphi_k I_{oz}}sin(\omega_{rf}t-\phi_k)]\} = \sum_{k=1}^{16}\{\sum_{o=1}^{2}\omega_o\left[I_{oz}\frac{2}{\omega_{rf}}sin(0.5\alpha)cos((k-0.5)\alpha-\phi_k) + e^{-i\varphi_k I_{oz}}I_{oy}e^{i\varphi_k I_{oz}}\frac{-2}{\omega_{rf}}sin(0.5\alpha)sin((k-0.5)\alpha-\phi_k)\right]\}.$$ Eqn. (S33)

If we define $(k-0.5)\alpha-\phi_k$ as Φ_k and to use ϕ_k values from the Table S3, then Φ_k for sixteen blocks can be summarized in the Table S5.

k	1	2	3	4	5	6	7	8	9	10	11	12	13	14	15	16
ϕ_k	0	2α	2α	4α	4α	6α	6α	8α	8α	10α	10α	12α	12α	14α	14α	16α
Φ_k	x	-x	x	-x	x	-x	x	-x	x	-x	x	-x	x	-x	x	-x

Table S5 The summary of ϕ_k and Φ_k phases. x = 0.5α.

According to Table S5, Eqn. (S33) can be rewritten as follows:

$$4T_R H^{(1)}_{ave,\sigma} = \sum_{k=1}^{16}\{\sum_{o=1}^{2} \omega_o \left[I_{oz}\frac{2}{\omega_{rf}} sin(0.5\alpha)cos(0.5\alpha) + e^{-i\varphi_k I_{oz}} I_{oy} e^{i\varphi_k I_{oz}} \frac{2(-1)^k}{\omega_{rf}} sin(0.5\alpha)sin(0.5\alpha)\right]\}. \quad \text{Eqn. (S34)}$$

If we take into account that:

$$\sum_{k=1}^{16} e^{-i\varphi_k I_{oz}} I_{oy} e^{i\varphi_k I_{oz}} (-1)^k = 0, \quad \text{Eqn. (S35)}$$

then Eqn. (S34) can be rewritten as:

$$4T_R H^{(1)}_{ave,\sigma} = \sum_{k=1}^{16}\{\sum_{o=1}^{2} \omega_o \left[I_{oz}\frac{2}{\omega_{rf}} sin(0.5\alpha)cos(0.5\alpha)\right]\} = \sum_{o=1}^{2} \frac{\omega_o}{\omega_{rf}} 16 sin(\alpha) I_{oz} = 16\frac{sin(\alpha)}{\nu_{rf}/\nu_R}\left[\Sigma\nu_{12}\frac{(I_{1z}+I_{2z})}{2} + \Delta\nu_{12}\frac{(I_{1z}-I_{2z})}{2}\right]T_R, \quad \text{Eqn. (S36)}$$

where $\Sigma\nu_{12} = \frac{\omega_1+\omega_2}{2\pi}$ and $\Delta\nu_{12} = \frac{\omega_1-\omega_2}{2\pi} = \nu_1 - \nu_2$.

The total FOH is the sum of the dipolar and chemical shift (Eqn. (S31) + Eqn. (S36)), and is:

$$4T_R H^{(1)}_{ave} = \{2I_{1z}I_{2z} - I_{1x}I_{2x} - I_{1y}I_{2y}\}3sin(2\alpha)sin^2(\beta)cos(2\gamma)(\nu_D T_R)\frac{\nu_{rf}/\nu_R}{\left(1-(\nu_{rf}/\nu_R)^2\right)} + 16\frac{sin(\alpha)}{\nu_{rf}/\nu_R}\left[\Sigma\nu_{12}\frac{(I_{1z}+I_{2z})}{2} + \Delta\nu_{12}\frac{(I_{1z}-I_{2z})}{2}\right]T_R. \quad \text{Eqn. (S37)}$$

Considering that: $[I_{1z}I_{2z}, I_{1x}I_{2x} + I_{1y}I_{2y}] = [(I_{1z} + I_{2z}), I_{1x}I_{2x} + I_{1y}I_{2y}] = 0$ and $I_{1z}I_{2z}$ and $(I_{1z} + I_{2z})$ terms commute with the initial and final operators (Eqn. S1)), then Eqn. (S37) can be modified as follows:

$$4T_R H^{(1)}_{ave} = -\{I_{1x}I_{2x} + I_{1y}I_{2y}\}3sin(2\alpha)sin^2(\beta)cos(2\gamma)(\nu_D T_R)\frac{\nu_{rf}/\nu_R}{\left(1-(\nu_{rf}/\nu_R)^2\right)} + 16\frac{sin(\alpha)}{\nu_{rf}/\nu_R}(\Delta\nu_{12}T_R)\frac{(I_{1z}-I_{2z})}{2}. \quad \text{Eqn. (S38)}$$

The final version of Eqn. (S37) can modified to express the Cartesian operators with fictitious spin-½ formalism[7]:

$$4T_R H_{ave}^{(1)} = -3sin(2\alpha_{rf})sin^2(\beta)cos(2\gamma)(\nu_D T_R)\frac{\nu_{rf}/\nu_R}{\left(1-(\nu_{rf}/\nu_R)^2\right)} I_x^{(2,3)} + 16\frac{sin(\alpha_{rf})}{\nu_{rf}/\nu_R}(\Delta\nu_{12}T_R) I_z^{(2,3)}, \quad \text{Eqn. (S39)}$$

where $\alpha_{rf} = \alpha = 0.5\pi\nu_{rf}T_R$.

The measured operator (Eqn. S1) in fictitious spin- ½ representation can be written as follows:

$$\langle I_{2z}\rangle(t_{mix}) = \int d\Omega\, Tr\{I_{2z}U_{tot}(t_{mix})I_{1z}U_{tot}^{-1}(t_{mix})\} \approx \int d\Omega\, Tr\left\{I_{2z}e^{-iN\left(4T_R H_{ave}^{(1)}\right)}I_{1z}e^{iN\left(4T_R H_{ave}^{(1)}\right)}\right\} = \int d\Omega\, Tr\left\{\left(I_z^{(1,4)} - I_z^{(2,3)}\right)e^{-iN\left(4T_R H_{ave}^{(1)}\right)}\left(I_z^{(1,4)} + I_z^{(2,3)}\right)e^{iN\left(4T_R H_{ave}^{(1)}\right)}\right\}. \quad \text{Eqn. (S40)}$$

Since Eqn. (S38) depends only on zero-quantum terms, Eqn. (S40) can be simplified as follows:

$$\langle I_{2z}\rangle(t_{mix}) = 0.5 - \int d\Omega\, Tr\left\{I_z^{(2,3)}e^{-iN\left(4T_R H_{ave}^{(1)}\right)}I_z^{(2,3)}e^{iN\left(4T_R H_{ave}^{(1)}\right)}\right\}. \quad \text{Eqn. (S41)}$$

The propagator, $e^{-iN\left(4T_R H_{ave}^{(1)}\right)}$, can be written as:

$$e^{-iN\left(4T_R H_{ave}^{(1)}\right)} = e^{-iN\left(a_z I_z^{(2,3)} - a_x I_x^{(2,3)}\right)} = e^{i\varphi_{zx}I_y^{(2,3)}}e^{-ir_{zx}NI_z^{(2,3)}}e^{-i\varphi_{zx}I_y^{(2,3)}}, \quad \text{Eqn. (S42)}$$

, where $r_{zx} = \sqrt{a_z^2 + a_x^2}$ and $\tan\varphi_{zx} = \frac{a_x}{a_z}$. The values a_x and a_z are:

$$a_x = 3sin(2\alpha_{rf})sin^2(\beta)cos(2\gamma)(\nu_D T_R)\frac{\nu_{rf}/\nu_R}{\left(1-(\nu_{rf}/\nu_R)^2\right)}, \quad a_z = 16\frac{sin(\alpha_{rf})}{\nu_{rf}/\nu_R}(\Delta\nu_{12}T_R). \quad \text{Eqn. (S43)}$$

Substituting Eqn. (S42) into Eqn. (S43), the final eqn. for the transferred signal is:

$$\langle I_{2z}\rangle(t_{mix} = N4T_R) = \int d\Omega \frac{a_x^2}{a_z^2 + a_x^2} sin^2\left(0.5N\sqrt{a_z^2 + a_x^2}\right). \quad \text{Eqn. (S44)}$$

Simulations and Experiments

Figure S1 shows the dipolar truncation[8] for MODIST with 101° (Figure S1B) and 45° (Figure S1C) flip angles, resulting from the presence of a third spin strongly dipolar-coupled to the initial spin. For the 22.5° flip angle (Figure S1D), the presence of the additional spin improves transfer efficiency within the simulated time period (red line compared to gray).

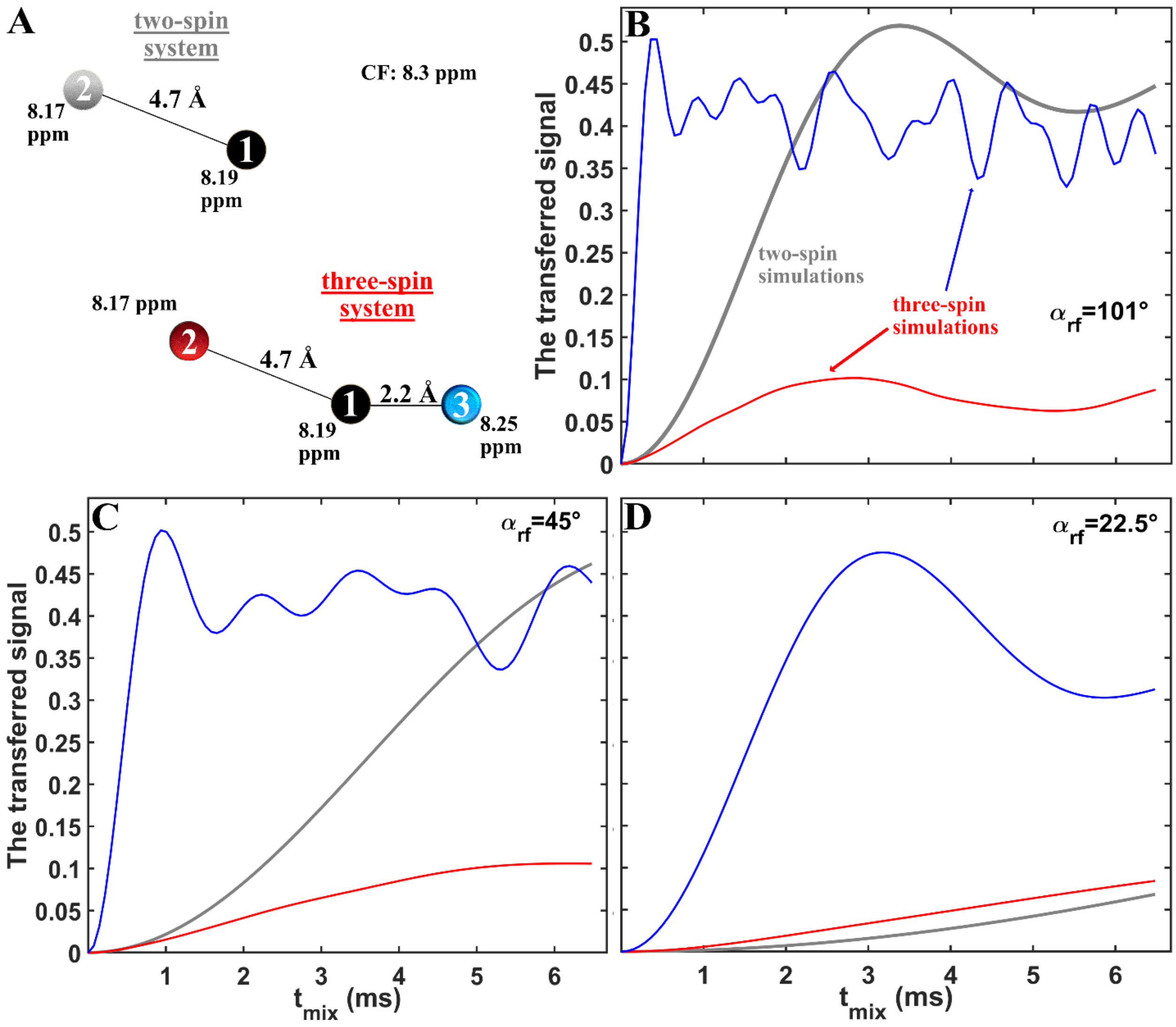

Figure S1 Transferred numerical MODIST signals were simulated for two-spin (gray) and three-spin (red and blue) systems with 101° (B), 45° (C), and 22.5° (D) flip angles. (A) schematically displays the simulated spin systems: the two-spin system (top left) and the three-spin system (bottom right). In (B)-(D), for the gray curve, the distance is 4.7 Å. For the red and blue curves, the red curve represents the transfer signal between the weaker dipolar-coupled spin pair with a distance of 4.7 Å ($I_{z1} \rightarrow I_{z2}$), while the blue curve represents the transfer signal between the stronger dipolar-coupled spin pair with a distance of 2.3 Å ($I_{z1} \rightarrow I_{z3}$). The isotropic chemical shifts for spins 1, 2, and 3 were 8.19, 8.17, and 8.25 ppm, respectively. All simulations used a 55.555 kHz MAS, an 8.3 ppm carrier frequency, and an 850 MHz ^{1}H Larmor frequency.

Figure S2-S3 shows the transfer to the second amide (Figure S2) and to aliphatic spins (in total, Figure S3) with 23° (A), 27° (B), 31 (C), 35 (D), 39 (E) and 43° (F) flip angles. All flip angles provide similar transfer efficiency for the multiple-spin system (orange), and the undesired amide-aliphatic transfer do not exceed 4%.

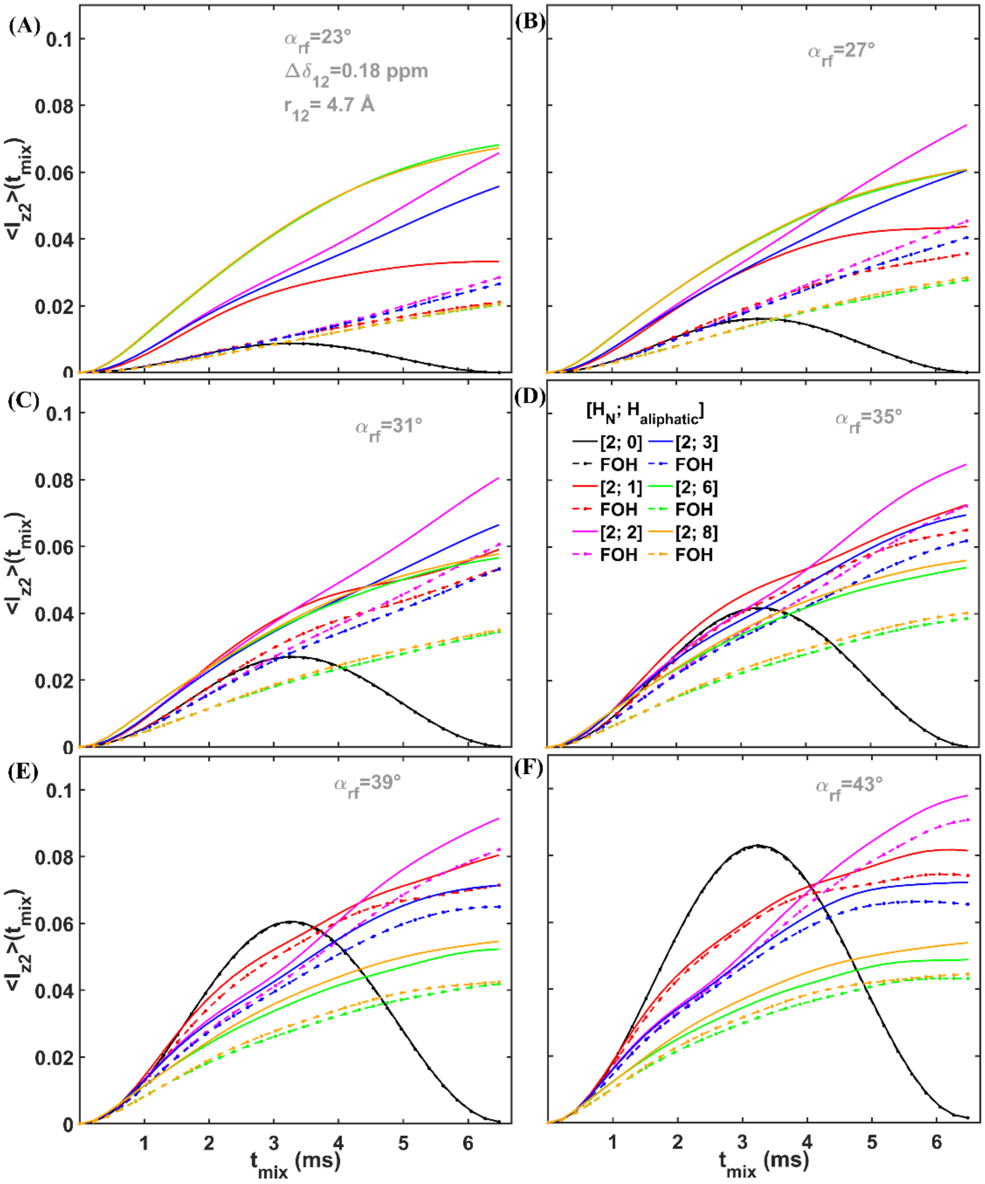

Figure S2 Numerical (solid) and FOH (dashed lines with points) analysis of MODIST was conducted for systems comprising up to ten spins, using six different flip angles: 23° (A), 27° (B), 31° (C), 35° (D), 39° (E) and 43° (F).

The transferred signal as a function of mixing time and the number of simulated spins [amide spins; aliphatic spins]: black – [2;0]; red – [3;0]; magenta – [4;0]; blue – [5;0]; green – [5;3]; orange – [5;5]. The distances between pairs of spins are shown in Table S1. The spatial coordinates of the ten spins were taken from the helical structure of Influenza A M2. The dipolar coupling values and the angles between all spin pairs (i,j) were calculated according to these coordinates. All simulations used 8.3 ppm carrier frequency and 850 MHz ^{1}H Larmor frequency.

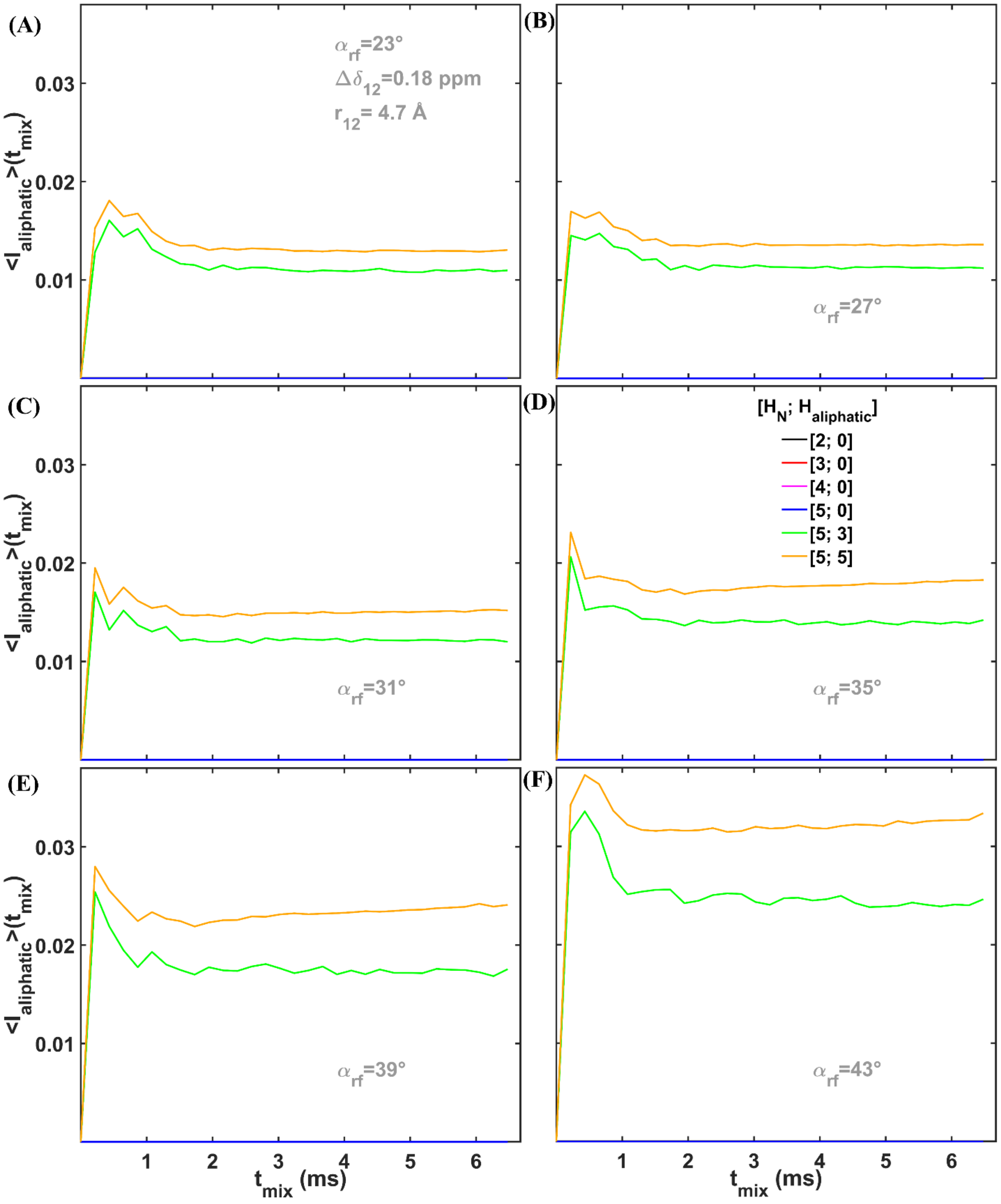

Figure S3 Numerical analysis of MODIST was conducted for systems comprising up to ten spins, using six different flip angles: 23° (A), 27° (B), 31° (C), 35° (D), 39° (E) and 43° (F). The total signal transferred to aliphatic spins as a function of mixing time and the number of simulated spins [amide spins; aliphatic spins]: black – [2;0];

red – [3;0]; magenta – [4;0]; blue – [5;0]; green – [5;3]; orange – [5;5]. The distances between pairs of spins are shown in Table S1. The spatial coordinates of the ten spins were taken from the helical structure of Influenza A M2. The dipolar coupling values and the angles between all spin pairs (i,j) were calculated according to these coordinates. All simulations used 8.3 ppm carrier frequency and 850 MHz ^{1}H Larmor frequency.

Figure S4 shows the polarization transfer (total amide and transferred signals) with 101° (A, D), 45° (B, E), and 22.5° (C, F) flip angles. A similar behavior to that observed under 55.555 kHz is noted, leading to nearly the same conclusions:

- For the ten-spin system, MODIST with all three flip angles provides a similar simulated transfer efficiency of around 5%.
- There is agreement between the numerical and FOH curves for MODIST with 101° and 45° flip angles.
- Facilitated dipolar recoupling appears and is preserved as the size of the simulated spin system increases.
- Better preservation of the total amide signal is observed for MODIST using 45° and 22.5° flip angles compared to 101°. This metric helps explain the good performance of the more selective sequences in experiments (where there are more than 10 spins).

However, one difference is observed: for the 22.5° flip angle at 110 kHz MAS, the transferred signal grows at around half the rate compared to simulations at 55.555 kHz, while the rates for the other flip angles remain unchanged. This is consistent with second-order effects becoming significant for 22.5° MODST.

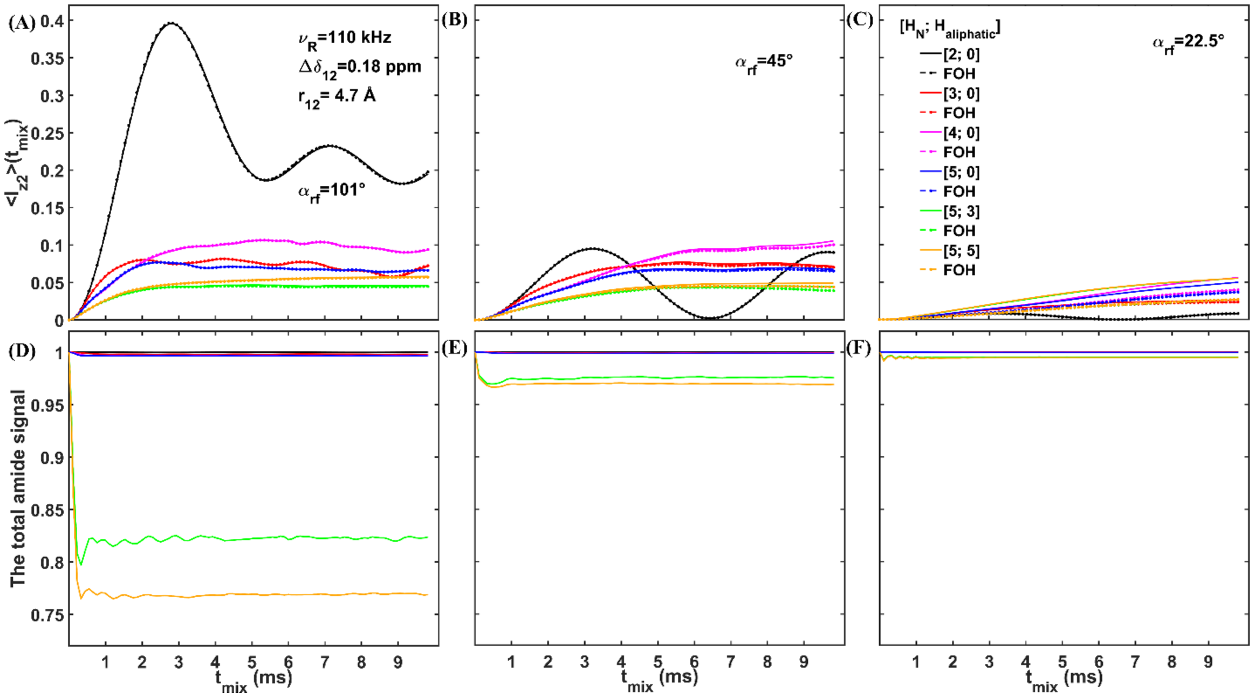

Figure S4 Numerical (solid) and FOH (dashed lines with points) analysis of MODIST at a 110 kHz MAS was conducted for systems comprising up to ten spins, using three different flip angles: 101° (B), 45° (C) and 22.5° (D). The transferred (A-C) and total amide signal (D-F, the signal of the spin 1 + the transferred signals to the rest of the amide spins) as functions of mixing time and the number of simulated spins [amide spins; aliphatic spins]: black – [2;0]; red – [3;0]; magenta – [4;0]; blue – [5;0]; green – [5;3]; orange – [5;5]. In all simulations, the initial signal was on spin 1 and the transferred signal was measured on spin 2. The distance and the isotropic chemical shift difference between spin 1 and spin 2 were 4.7 Å and 0.18 ppm, respectively. The distances between pairs of spins are shown in Table S1. The spatial coordinates of the ten spins were taken from the helical structure of Influenza A M2. The dipolar coupling values and the angles between all spin pairs (i,j) were calculated according to these coordinates. All simulations used 8.3 ppm carrier frequency and 850 MHz ^{1}H Larmor frequency.

Figure S5A compares 1D (HNH)H^{MODIST} spectra of WT M2 as a function of mixing time and flip angle. MODIST with α_{rf} =22.5° (black) provides the highest preservation of total amide signal compared to α_{rf} =45° (blue) and α_{rf} =101° (pink). For α_{rf} =101° (pink), the loss of the total amide signal is drastic and only a small portion of the total starting amide signal

remains; with a 4.608 ms mixing time, the ratio of peak intensities in the amide region is 1:0.75:0.13 for black (22.5°), blue (45°) and pink (101°).

Figure S5C compares 2D (H)N(H)H^{MODIST} spectra with three different flip angles (22.5°, 45° and 67.5°) at a 3.456 ms mixing time. Even with a flip angle of 67.5° (red), MODIST preserves the total amide signal substantially worse than 22.5° (black) or 45° (blue). While these 2D experiments do not provide enough resolution to distinguish the correlations between different amide protons in this sample, the correlation between $H37_A$ and $W41_B$ (schematically shown in Figure S5B and labeled with '1' in Figure S5C) is well resolved. The distance and the offset difference between $H37_A$ and $W41_B$ are about 2.6 Å and 0.94 ppm, respectively based on the structure with a PDB ID 2N70.[9] Figure S5D1 shows the intensity of the $H37_A$-$W41_B$ correlation as a function of mixing time. Despite a large chemical shift difference between the two amide protons (0.94 ppm), the build-up curves are observed for all three flip angles. However, MODIST with 45° flip angle (blue) provides the highest transfer efficiency of the three flip angles tested. For 22.5°, the correlation is slightly oscillatory above the noise level. The evaluation of another cross-peak (Figure S5D2, labeled as '2' in Figure S5C) shows that in another case, MODIST with 22.5° (black) flip angle outperforms the MODIST with 45° (blue) flip angle and 67.5° (red) flip angle.

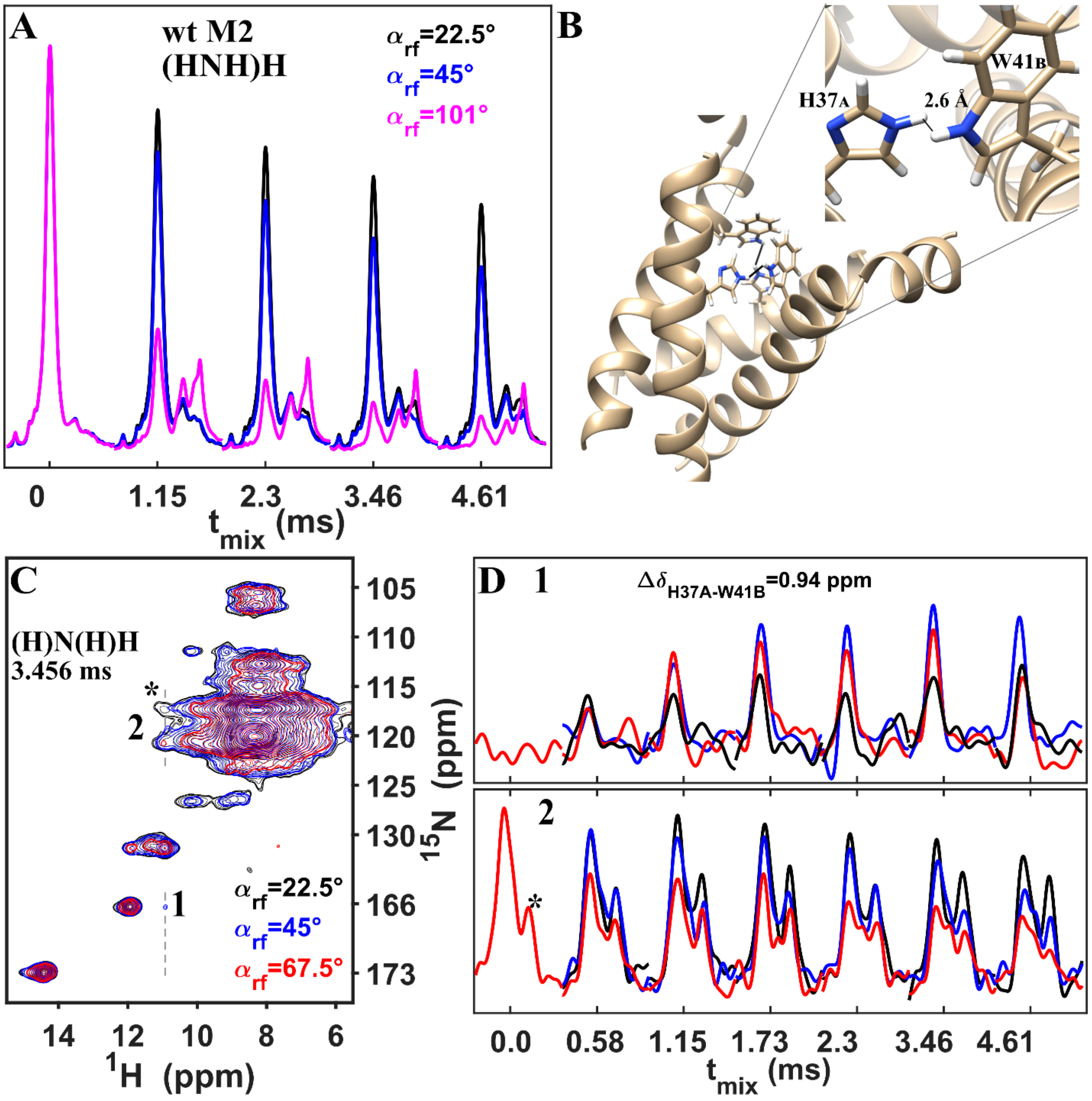

Figure S5 (H)N(H)H^{MODIST} spectra of WT influenza A M2 acquired with different flip angles. (A) 1D (HNH)H spectra as a function of mixing time and flip angles: 101° - pink; 45° - blue and 22.5° - black. (B) The structure of influenza A M2 is based on PDB 2N70.[9] (C) 2D (H)N(H)H^{MODIST} spectra at a 3.456 ms mixing time with flip angles of 67.5° - red; 45° - blue and 22.5° - black. (D)1-2 Two vertical slices from (H)N(H) H^{MODIST} spectra, recorded at seven different mixing times. Data were recorded at a 600 MHz spectrometer with 55.555 kHz MAS. The proton carrier frequency was set to 3 ppm. Further experimental details are provided below in Figures S7A and S10.

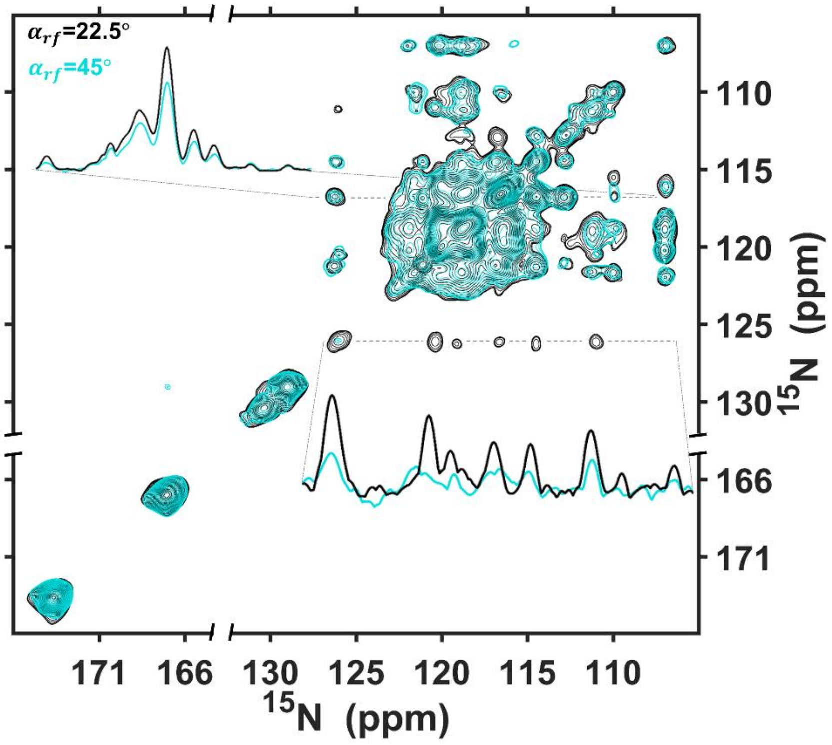

Figure S6 ^{15}N – ^{15}N projections of 3D (H)N(H)(H)NH^{MODIST} S31N M2 spectra with 22.5° (black) and 45° (cyan) flip angle. Data were recorded at a 600 MHz spectrometer with 55.555 kHz MAS. In both experiments 5.76 ms MODIST mixing was applied. The proton carrier frequency was set to 1.7 ppm. Further experimental details are provided below in Figures S7A and S13.

Figure S7 compares 2D (H)C(H)(H)C spectra recorded on a sample of WT M2 at a 600 MHz spectrometer , showcasing the performance of MODIST with 22.5° (black) and 33.75° (magenta) flip angles (A), along with spectra recorded at a 1200 MHz spectrometer using an S31N M2 sample, demonstrating the performance of MODIST with 22.5° (black) and 45° (red) (B). In both cases MODIST with 22.5° exhibits superior efficiency for aliphatic-aliphatic contacts compared with the other two flip angles.

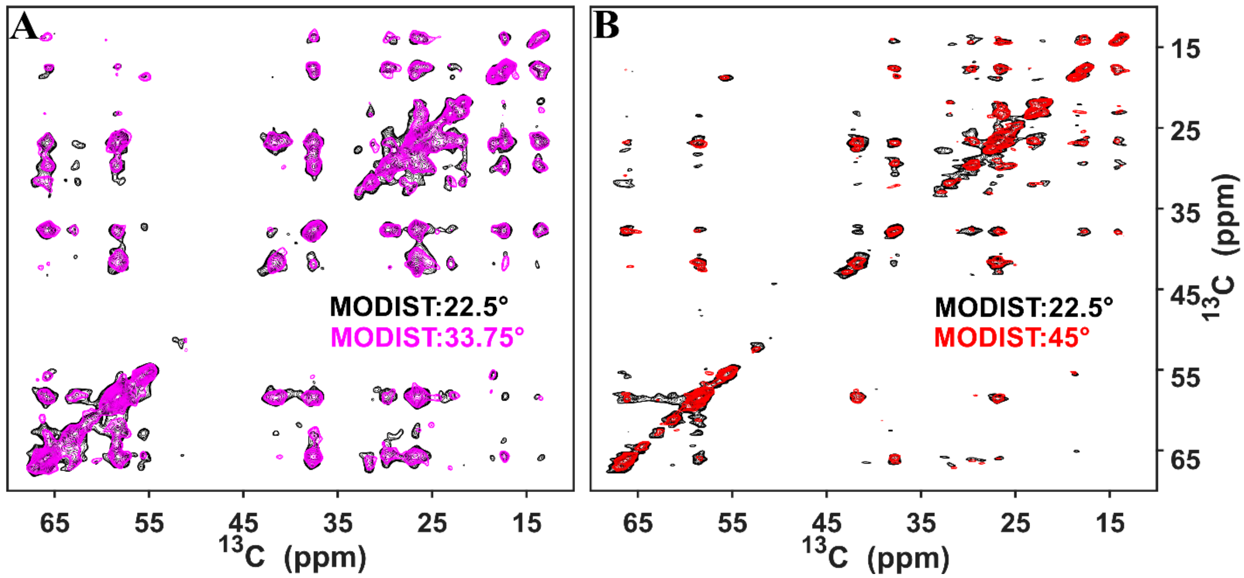

Figure S7 2D (H)C(H)(H)C^{MODIST} experiments were recorded at a 600 MHz spectrometer with a sample of WT M2 (A) and at a 1200 MHz spectrometer with a sample of S31N M2 (B). (A) MODIST element with 22.5° - black and 33.75° - magenta. The mixing time was 4.608 ms. (B) MODIST element with 22.5° - black and 45° - red. The mixing time was 6.336 ms. All experiments were performed with 55.555 kHz MAS. The proton carrier frequency was set to 1 ppm. For (A), further experimental details are provided below in Figures S7B and Figure S11.

Figure S8 compares the ^{13}C-^{13}C projection from a 3D (H)C(H)(H)CH spectrum (black) with a 2D (H)CC spectrum in which 6.912 ms RFDR was applied for ^{13}C mixing. Additional black peaks show inter-residue correlations that are not observed with RFDR, which primarily excites correlations within a residue. Conversely, additional red peaks show intra-residue correlations that are not observed with MODIST.

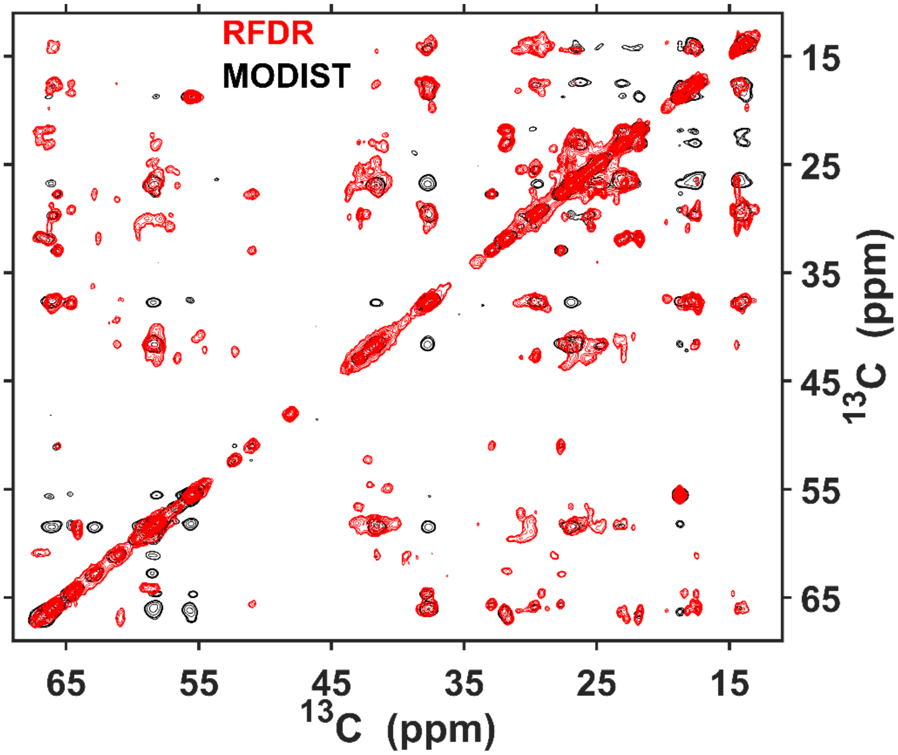

Figure S8 2D (H)CC experiment with RFDR element (red, 6.91 ms mixing, 83.333 kHz rf-field strength, 35 ppm carrier frequency position) and ^{13}C-^{13}C projection from 3D (H)C(H)(H)CH experiment with MODIST element (black, 5.76 ms mixing and 22.5° flip angle) were collected at a 1200 MHz spectrometer with an S31N M2 sample. Further experimental details are provided below in Figures S8A and S16 for RFDR and Figure S8B and Figure S15 for MODIST.

Experimental Methods

Simulations

MODIST simulations were performed using in-house MATLAB scripts based on the numerical solution of the equation of motion.[10] The following table summarizes all distances (r_{ij}) and orientations (θ_{ij}, ϕ_{ij}) between each pair of spins (i,j) (shown below), used in simulations:

"(1,2)|4.7 Å|(0°,0°)"	"(2,9)|6.4 Å|(153°,80°)"	"(5,6)|4.5 Å|(71°,14°)"
"(1,3)|2.8 Å|(55°,0°)"	"(2,10)|6.6 Å|(149°,12°)"	"(5,7)|5.4 Å|(46°,76°)"
"(1,4)|2.8 Å|(117°,85°)"	"(3,4)|4.3 Å|(131°,51°)"	"(5,8)|4.2 Å|(85°,32°)"

"(1,5)|4.1 Å|(134°,37°)"	"(3,5)|4.7 Å|(159°,88°)"	"(5,9)|5.2 Å|(70°,68°)"
"(1,6)|2.3 Å|(124°,21°)"	"(3,6)|5.1 Å|(125°,10°)"	"(5,10)|3.3 Å|(55°,67°)"
"(1,7)|2.7 Å|(69°,55°)"	"(3,7)|2.3 Å|(106°,67°)"	"(6,7)|4.8 Å|(62°,40°)"
"(1,8)|2.8 Å|(152°,22°)"	"(3,8)|5.4 Å|(139°,8°)"	"(6,8)|1.8 Å|(131°,65°)"
"(1,9)|3.1 Å|(110°,80°)"	"(3,9)|4.3 Å|(128°,58°)"	"(6,9)|4.2 Å|(86°,57°)"
"(1,10)|3.6 Å|(105°,12°)"	"(3,10)|2.8 Å|(153°,34°)"	"(6,10)|5.4 Å|(86°,15°)"
"(2,3)|3.9 Å|(144°,0°)"	"(4,5)|2.7 Å|(125°,20°)"	"(7,8)|4.6 Å|(138°,30°)"
"(2,4)|6.5 Å|(157°,85°)"	"(4,6)|2.7 Å|(91°,41°)"	"(7,9)|2.4 Å|(149°,40°)"
"(2,5)|8.1 Å|(159°,37°)"	"(4,7)|5.2 Å|(65°,75°)"	"(7,10)|3 Å|(129°,34°)"
"(2,6)|6.3 Å|(162°,21°)"	"(4,8)|3.6 Å|(110°,64°)"	"(8,9)|3.2 Å|(64°,53°)"
"(2,7)|4.5 Å|(146°,55°)"	"(4,9)|5.3 Å|(88°,87°)"	"(8,10)|4.9 Å|(71°,3°)"
"(2,8)|7.3 Å|(169°,22°)"	"(4,10)|4.5 Å|(86°,46°)"	"(9,10)|3.6 Å|(88°,36°)"

Table S6 The used distances (r_{ij} in Å) and the orientations (θ_{ij}, ϕ_{ij} in °) between each pair of spins (i,j) up to ten-are shown as "$(i, j)|r_{ij}|(\theta_{ij}, \phi_{ij})$".

Sample Preparation

Influenza A wild type M2 and S31N M2 proteins, residues 18-60, were prepared according to the protocols in the references [11,12]. The samples were packed into Bruker 1.3 mm rotors via centrifugation. The samples contained Cu^{2+} ethylenediaminetetraacetic acid to accelerate the acquisition.

Solid state NMR spectroscopy

The required rf-field powers of MODIST pulses were calculated according to the calibrated ^{1}H 90°-pulse. During MODIST pulses the ^{1}H carrier frequency was set to 3 ppm and 1 ppm for excitation of amide proton-proton and aliphatic proton-proton cross peaks, respectively.

As previously shown, placing the CF in aliphatic region slightly improves the performance of MODIST (Figure S6-S8 in Ref.[5]).

Figures S9 shows 2D (H)N(H)H (A) and 3D (H)N(H)(H)NH (B) sequences used with a MODIST element for the excitation of amide proton-proton cross peaks.

Figure S10A shows 2D (H)CC sequence used with RFDR element for the excitation of carbon-carbon correlations. Figure S10B shows 2D (H)C(H)(H)C sequence used with a MODIST element for exciting aliphatic proton-proton cross peaks.

Figure S11 shows 3D (H)C(H)(H)CH sequences used with a MODIST element for the excitation of aliphatic proton-proton cross peaks.

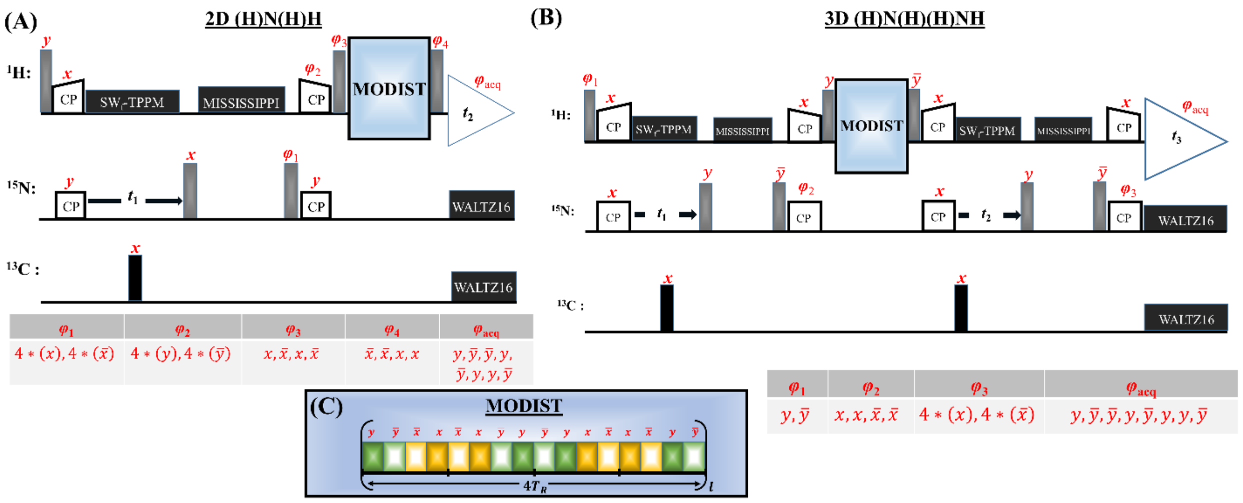

Figure S9 2D (H)N(H)H (A) and 3D (H)N(H)(H)NH (B) sequences. (C) The repeated MODIST block, consisting of 16 pulses with $0.25T_R$ of the width and α_{rf} flip angle for a single pulse. π/2-pulses are indicated by light, π-pulses by dark grey rectangles. All phase cycling is shown in Figure. The ramped CP transfers from proton to nitrogen as well as from nitrogen to proton are indicated with a constant power on the nitrogen channel and a ramp in power on the proton channel. During the inderect dimensions (t_1) and (t_2), SW_f-TPPM decoupling[13] is applied. A single π-pulse in the middle of t_1 and t_2 decouples carbon-nitrogen interactions. Water supression is implemented with the MISSISSIPI sequence.[14] During acquisition, WALTZ16 decoupling[15] is applied on nitrogen and carbon channels.

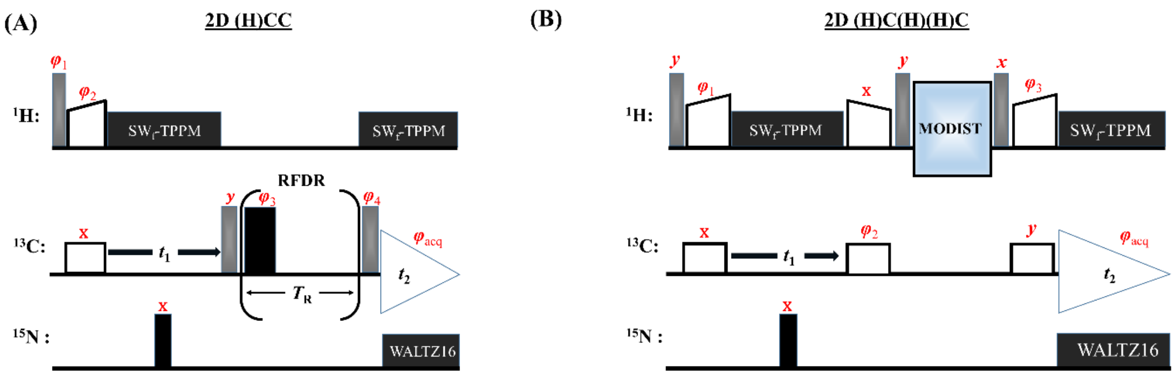

φ_1	φ_2	φ_3	φ_4	φ_{acq}
$y, \bar{y}$	$x, x, \bar{x}, \bar{x}$	$x, y, x, y, y, x, y, x, \bar{x}, \bar{y}, \bar{x}, \bar{y}, \bar{y}, \bar{x}, \bar{y}, \bar{x}$	$4 * (\bar{y}), 4 * (y)$	$y, \bar{y}, \bar{y}, y, \bar{y}, y, y, \bar{y}$

φ_1	φ_2	φ_3	φ_{acq}
$x, \bar{x}$	$x, x, \bar{x}, \bar{x}$	$4 * (y), 4 * (\bar{y})$	$\bar{x}, x, x, \bar{x}, x, \bar{x}, \bar{x}, x$

Figure S10 2D (H)CC (A) and 2D (H)C(H)C (B) sequences. (A) RFDR with XY16 phase cycling[16] was applied. (B) MODIST element with 22.5° flip angle was applied. π/2-pulses are indicated by light and π-pulses by dark grey rectangles. All phase cycling is shown in the figure. The ramped CP transfers from proton to carbon as well as from carbon to proton are indicated with constant power on the carbon channel and a ramp in power on the proton channel. During the inderect dimension (t_1) and detection, SW$_f$-TPPM decoupling[13] is applied on proton channel. A single π-pulse in the middle of t_1 and t_2 decouples carbon-nitrogen interactions. During acquisition, WALTZ16 decoupling[15] is applied on nitrogen channel.

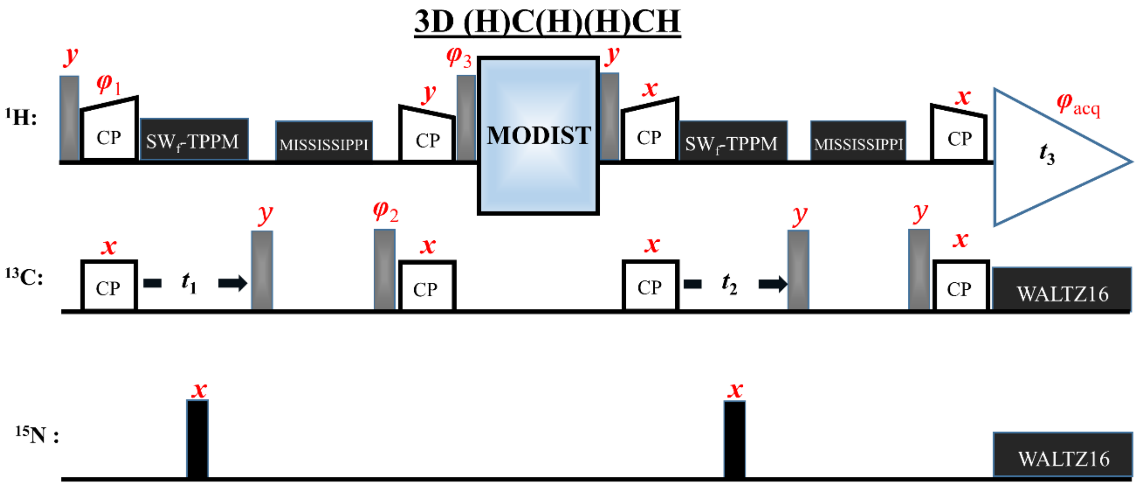

φ_1	φ_2	φ_3	φ_{acq}
$x, \bar{x}$	$4 * (y), 4 * (\bar{y})$	$2 * (x), 2 * (\bar{x})$	$y, \bar{y}, \bar{y}, y, \bar{y}, y, y, \bar{y}$

Figure S11 3D (H)C(H)(H)CH (B) sequence. π/2-pulses are indicated by light and π-pulses by dark grey rectangles. All phase cycling is shown in figure. The ramped CP transfers from proton to carbon as well as from carbon to proton are indicated with constant power on the carbon channel and a ramp in power on the proton channel. During the inderect dimensions (t_1) and (t_2), SW_f-TPPM decoupling[13] is applied. A single π-pulse in the middle of t_1 and t_2 decouples carbon-nitrogen interactions. Water supression is implemented with the MISSISSIPI sequence.[14] During acquisition, WALTZ16 decoupling[15] is applied on nitrogen and carbon channels.

600 MHz: 2D (H)N(H)H, 3D (H)N(H)(H)NH and 2D (H)C(H)(H)C experiments were acquired on a Bruker Avance III HD spectrometer operating at 14.1 T (600 MHz ^{1}H frequency) using a DVT600W2 BL1.3 mm HXY probe. The experiments were performed at 55.555 kHz MAS, the temperature of the nitrogen cooling gas set to 243 K, with 1000 to 1300 liters per hour. For decoupling of the heteronuclear dipolar interactions SW_f-TPPM,[13] was used on the proton channel, and WALTZ-16[15] was used on heteronuclear channels. MISSISSIPPI[14] water suppression was applied. In the figures shown below: SW – spectral width; TD – the size of FID; IN_F –increment time; AQ – the acquisition time. 4 Dummy Scans were used.

2D (H)N(H)H

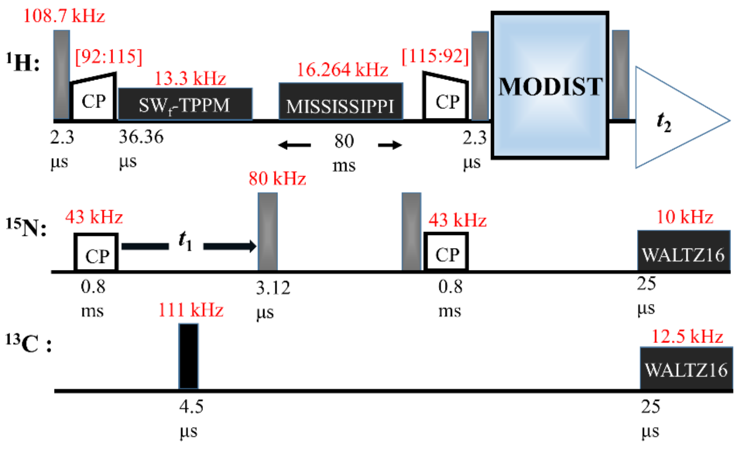

	^{1}H	^{15}N
**TD**	2048	400
**SW(ppm)**	40.1248	160
**IN_F(µs)**		102.94
**AQ(s)**	0.0425984	0.0205888

Number Scans: 24 | Recycle delay: 0.6 s |
Total Time of the experiment: 1.98 hours

Figure S12 The 2D (H)N(H)H spectrum and the experimental parameters used for measurement on Influenza A WT M2 (**the data is shown in Figure S5**). MODIST was applied for amide proton-proton recoupling. Red numbers represent rf-field power in kHz. Rectangle brackets indicate the minimal and maximal rf-field of ramped CPs. For

$^1H \rightarrow ^{15}N$ transfer an [80%:100%] linear ramp was used. For $^{15}N \rightarrow ^1H$ transfer a [100%:80%] linear ramp was used. The widths of the hard pulses are in µs, while the total duration of CP and decoupling are in ms. MODIST element was used for dipolar recoupling. During MODIST element proton carrier frequency was set to 3 ppm.

2D (H)C(H)(H)C

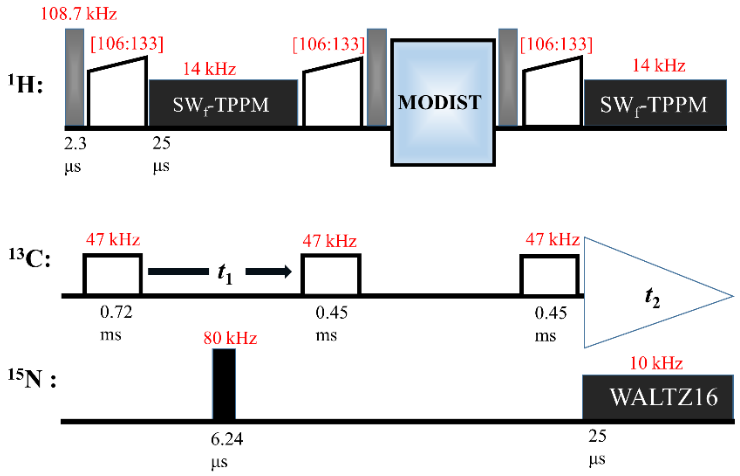

	^{13}C	^{13}C
**TD**	2048	700
**SW(ppm)**	553.1711	320
**IN_F(µs)**		20.74
**AQ(s)**	0.012288	0.0072604

Number Scans: 280 | Recycle delay: 0.4 s |
Total Time of the experiment: 16.53 hours

Figure S13 The 2D (H)C(H)(H)C spectrum and the experimental parameters used for measurement on Influenza A WT M2 (**the data is shown in Figure S7A**). MODIST was applied for aliphatic proton-proton recoupling. Red numbers represent rf-field power in kHz. Rectangle brackets indicate the minimal and maximal rf-field of ramped CPs. For $^1H \rightarrow ^{13}C$ and $^{13}C \rightarrow ^1H$ transfers [70%:100%] linear ramps were used. The widths of the hard pulses are in µs, while the total duration of CP and decoupling are in ms. MODIST element was used for dipolar recoupling. During MODIST element proton carrier frequency was set to 1.7 ppm.

3D (H)N(H)(H)NH

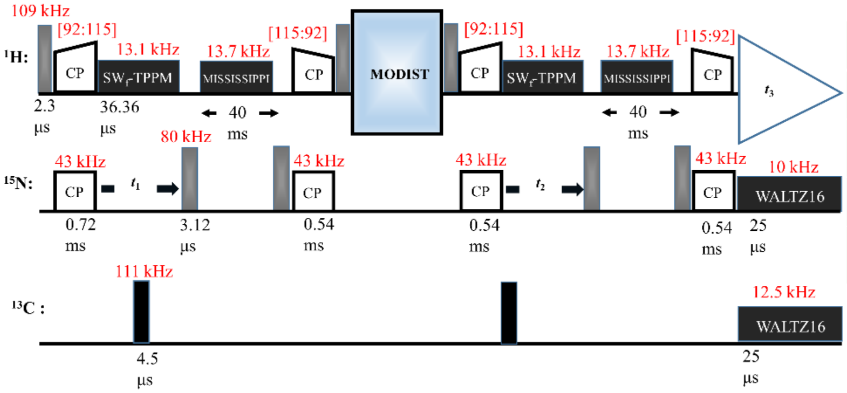

	^1H	^{15}N	^{15}N
**TD**	2048	190	166
**SW(ppm)**	40.1248	110	120
**IN_F(µs)**		149.73	137.26
**AQ(s)**	0.0425984	0.0143348	0.0113923

Number Scans: 8 | Recycle delay: 0.6 s |
Total Time of the experiment: 1.769 days

Figure S14 The 3D (H)N(H)(H)NH experiment with the experimental parameters used for measuring WT M2 (**the data is shown in Figure 10 in the main text**). MODIST was applied for amide proton-proton recoupling. Red numbers represent rf-field power in kHz. Rectangle brackets indicate the minimal and maximal rf-field of ramped CPs. For $^{1}H \rightarrow ^{15}N$ transfer an [80%:100%] linear ramp was used. For $^{15}N \rightarrow ^{1}H$ transfer a [100%:80%] linear ramp was used. The width of the hard pulses are in µs, while total duration of CP and decoupling are in ms. MODIST was used for dipolar recoupling.

850 MHz: 3D (H)N(H)(H)NH experiments were acquired on a Bruker Avance III spectrometer operating at 19.97 T (850 MHz ^{1}H field strength), equipped with a 1.3 mm HCN MAS probe at 55.555 kHz MAS. The temperature of the nitrogen cooling gas was set to 245 K. Heteronuclear dipolar interactions were decoupled with SW_f-TPPM,[13] (proton channel) and WALTZ-16,[15] (nitrogen channel). For water suppression, MISSISSIPPI[14] was used. In all the figures shown below: SW – spectral width; TD – the size of FID; IN_F –increment time; AQ – the acquisition time. 8 Dummy Scans were used.

3D (H)N(H)(H)NH

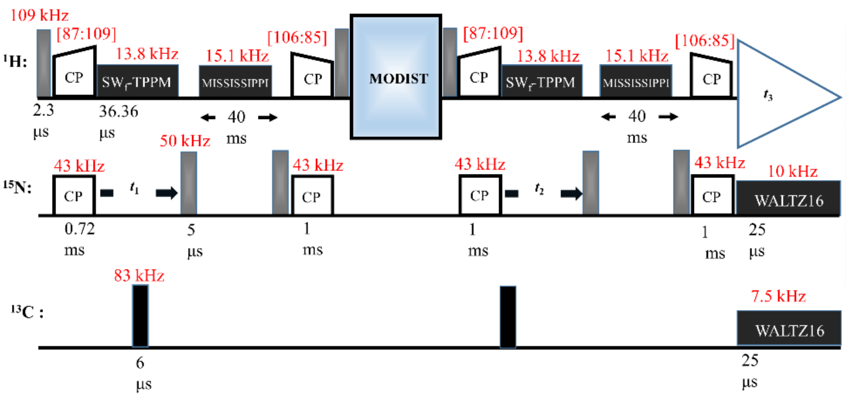

	^{1}H	^{15}N	^{15}N
TD	1280	200	200
SW(ppm)	30.4694	90	90
IN_F(µs)		128.9483	128.9483
AQ(s)	0.024704	0.0128948	0.0128948

Number Scans: 4 | Recycle delay: 0.4 s | Total Time of the experiment: 23.5 hours

Figure S15 The 3D (H)N(H)(H)NH experiment with the experimental parameters used for measuring WT M2 (**the data is shown in Figure S6**). MODIST was applied for amide proton-proton recoupling. Red numbers represent rf-field power in kHz. Rectangle brackets indicate the minimal and maximal rf-field of ramped CPs. For $^{1}H \rightarrow ^{15}N$ transfer an [80%:100%] linear ramp was used. For $^{15}N \rightarrow ^{1}H$ transfer a [100%:80%] linear ramp was used. The width

of the hard pulses are in μs, while total duration of CP and decoupling are in ms. MODIST was used for dipolar recoupling.

1200 MHz: 3D (H)N(H)(H)NH, 2D (H)C(H)(H)C and 2D (H)CC spectra were acquired on a Bruker Avance NEO spectrometer operating at 28.18 T (1200 MHz ^{1}H frequency) using a 1.3 mm HCN probe. The experiments were performed at 55.555 kHz MAS, the temperature of the nitrogen cooling gas is set to 245 K using 1000 liters per hour of flow. For decoupling of the heteronuclear dipolar interactions and water suppression SW$_f$-TPPM,[13] WALTZ-16[15] and MISSISSIPPI[14] were applied. In the Figures shown below: SW – spectral width; TD – the size of FID; IN_F –increment time; AQ – the acquisition time. 8 and 2 Dummy Scans were used for 3D and 2D experiments, respectively.

3D (H)C(H)(H)CH

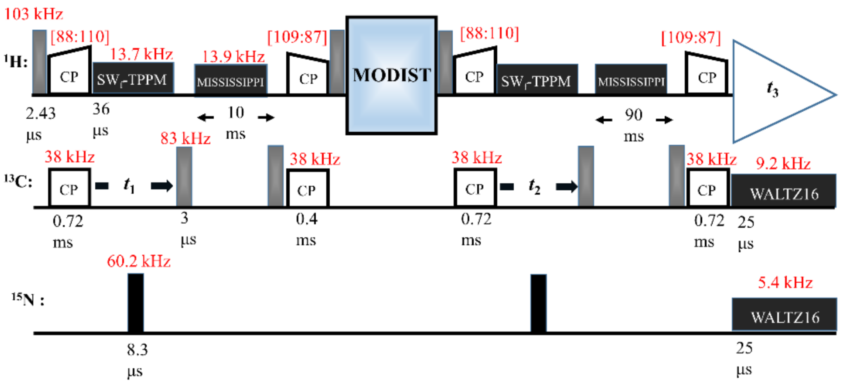

	^{1}H	^{13}C	^{13}C
**TD**	2048	200	424
**SW(ppm)**	40.4451	72.0295	118.3342
**IN_F(μs)**		46	28
**AQ(s)**	0.0210944	0.0046	0.005936

Number Scans: 8 | Recycle delay: 0.3 s | Total Time of the experiment: 3.41 days

Figure S16 The 3D (H)C(H)(H)CH experiment with the experimental parameters used for measuring S31N M2 (**the data is shown in Figure 11 in the main text and in Figure S6, red**). MODIST was applied for aliphatic proton-proton recoupling. Red numbers represent rf-field power in kHz. Rectangle brackets indicate the minimal and maximal rf-field of ramped CPs. For ^{1}H→^{13}C transfer an [80%:100%] linear ramp was used. For ^{13}C→^{1}H transfer a [100%:80%] linear ramp was used. The widths of the hard pulses are in μs, while the total duration of CP and decoupling are in ms. MODIST was used for dipolar recoupling.

2D (H)C(H)(H)C

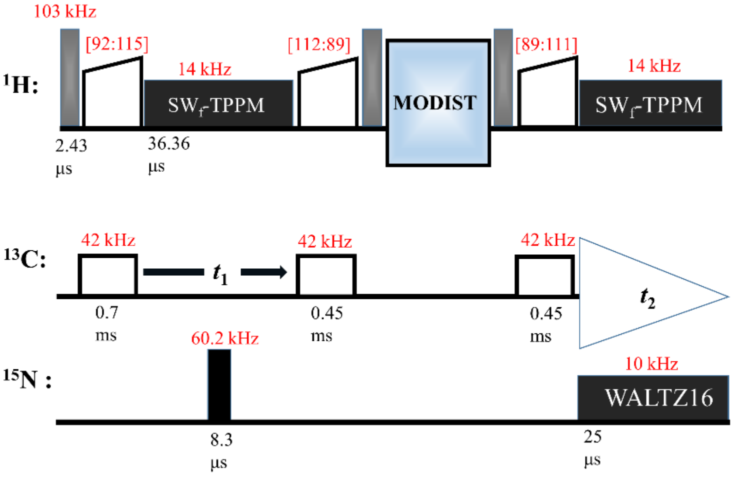

	^{13}C	^{13}C
TD	4410	2600
SW(ppm)	414.1697	320
IN_F(µs)		10.3542
AQ(s)	0.012288	0.0134605

Number Scans: 40 | Recycle delay: 0.3 s |
Total Time of the experiment: 9.6 hours

Figure S17 The 2D (H)C(H)(H)C spectrum and the experimental parameters used for measurement on Influenza A WT M2 (**the data is shown in Figure S7B**). MODIST was applied for aliphatic proton-proton recoupling. Red numbers represent rf-field power in kHz. Rectangle brackets indicate the minimal and maximal rf-field of ramped CPs. For ^{1}H→^{13}C transfer an [80%:100%] linear ramp was used. For ^{13}C→^{1}H transfer a [100%:80%] linear ramp was used. The widths of the hard pulses are in µs, while the total duration of CP and decoupling are in ms.

2D (H)CC

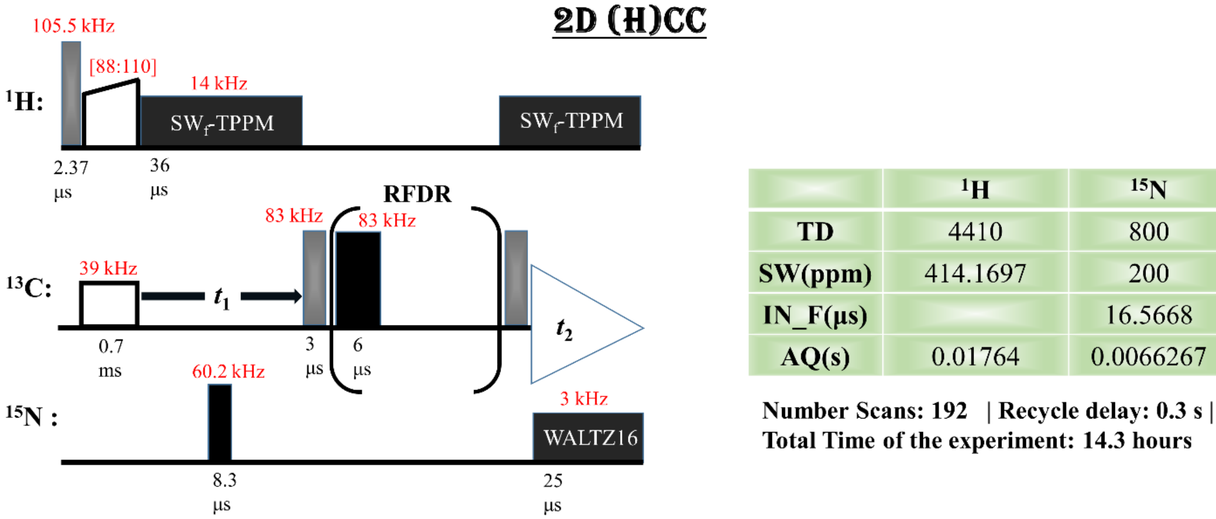

	^{1}H	^{15}N
TD	4410	800
SW(ppm)	414.1697	200
IN_F(µs)		16.5668
AQ(s)	0.01764	0.0066267

Number Scans: 192 | Recycle delay: 0.3 s |
Total Time of the experiment: 14.3 hours

Figure S18 The 2D (H)C(H)(H)C spectrum and the experimental parameters used for measurement on Influenza A WT M2 (**the data is shown in Figure S8**). RFDR was applied for carbon-carbon recoupling. Red numbers represent rf-field power in kHz. Rectangle brackets indicate the minimal and maximal rf-field of ramped CPs. For ^{1}H→^{13}C

transfer an [80%:100%] linear ramp was used. The width of the hard pulses are in μs, while total duration of CP and decoupling are in ms.

BRUKER PULSE PROGRAMS

The width of MODIST pulses is automatically calculated using the 'cnst31' parameter (the MAS rate in Hz). 'cnst19' defines the position of the ^{1}H carrier frequency during MODIST sequence (in ppm).

3D (H)C(H)(H)CH

```
;hChhCH MODIST with 0.25*MAS rf-field strength
; modified in the group of Dr. Loren Andreas

;parameters:
;p1 : 13C 90 pulse for 180 in t1
;pl3 : 13C power for 180
;p3 : 90 pulse at pl3
;p15 : contact time at pl1 (f1) and pl2 (f2)
;pl1 : power for p1
;pl20: 13C power for HC/CH transfers
;sp0 : proton power level during contact
;pl2 : =120dB, not used
;pl12 : decoupling power level (if not pl13)
;pl13 : special decoupliong power level
;d1 : recycle delay
;cnst21 : on resonance, usually = 0
;pcpd2 : pulse length in decoupling sequence
;pcpd4 : pulse length for X dec during acq
;cpdprg2 : cw, tppm (at pl12), or lgs, cwlg. cwlgs (LG-decoupling
;spnam0 : use e.g. ramp.100 for variable amplitude CP
```

```
;here pl13 is used instead of pl12)
;zgoptns : -Dfslg, -Dlacq, or blank


;pl11 : MODIST 0.25*MAS rate
;l21 : Number of MODIST blocks

;$COMMENT=basic cp experiment, arbitrary contact and decoupling schemes
;$CLASS=Solids
;$DIM=1D
;$TYPE=cross polarisation
;$SUBTYPE=simple 1D
;$OWNER=Bruker
prosol relations=<solids_cp>

#include <Avancesolids.incl>

;cnst20 : RF field achieved at pl13
;cnst21 : on resonance, usually = 0
;cnst22 : positive LG offset
;cnst23 : negative LG offset
;cnst24 : additional LG-offset
;cnst28 : carrier offset for water
;cnst11 : to adjust t=0 for acquisition, if digmod = baseopt
"acqt0=1u*cnst11"
"in0=inf1"
"in30=inf1"
"in10=inf2"
"d0=0"
"d10=0"
```

```
;aqseq 321

; 15N settings
"pcpd2=25"                    ;does not work!
"plw2=plw7"
"plw16=plw2*(pow(p7/25,2))"     ; 15N waltz 10kHz decoupling power level
"spoal2=0.5"   ; default value (irrelevant)
"spoff2=0.0"     ; on-resonance

 ; 13C settings
"plw17=plw3*(pow(p3/25,2))"     ; 13C waltz 10kHz decoupling power level

"d31=1s/cnst31" ;allow protection for misset l31


"p11 = (d31/4)"
"l22=16"

define delay mix
"mix = (l21*(l22*p11))"

1 ze
  mix
  1u fq=cnst21:f1
2 d1 do:f2 do:f3

  (p1  pl1 ph7):f1
  (p15 pl20 ph2):f3 (p15:sp0 ph10):f1
  1u cpds1:f1
   (center (d0) (p7*2 ph0 pl7):f2)
```

```
  1u do:f1

  (p3 pl3 ph1):f3
  1u fq=cnst28:f1
 (p30*0.025 pl13 ph0):f1
 (p30*0.025 pl13 ph1):f1
 (p30*0.025 pl13 ph0):f1
 (p30*0.025 pl13 ph1):f1
  1u fq=cnst21:f1
  (p3 pl3 ph11):f3

  (p17 pl20 ph4):f3 (p17:sp10 ph3):f1

  (p1  pl1 ph5):f1

1u fq=cnst19(ppm):f1
 ;;;;;;;;;;;;;;; MODIST block starts
3
4 (p11 pl11 ph8^):f1
  lo to 4 times l22
  lo to 3 times l21
;;;;;;;;;;;;;;; MODIST block ends
  1u fq=cnst21(ppm):f1

 (p1  pl1 ph6):f1

  (p17 pl20 ph12):f3 (p17:sp0 ph20):f1
  1u cpds1:f1
   (center (d10) (p7*2 ph0 pl7):f2)
  1u do:f1
```

```
 (p3 pl3 ph15):f3
 1u fq=cnst28:f1
(p30*0.225 pl13 ph0):f1
(p30*0.225 pl13 ph1):f1
(p30*0.225 pl13 ph0):f1
(p30*0.225 pl13 ph1):f1
 1u fq=cnst21:f1
 (p3 pl3 ph16):f3

 (p17 pl20 ph13):f3 (p17:sp10 ph14):f1

 1u cpds2:f2 cpds3:f3

 go=2 ph31
 1m do:f2     do:f3
 10m mc #0 to 2
 F1PH(calph(ph2, +90), caldel(d0, +in0))   ;first 13C dim
 F2PH(calph(ph12, +90), caldel(d10, +in10))  ;second 13C dim


HaltAcqu, 1m      ;jump address for protection files
exit              ;quit

ph0=0
ph1=1
ph11 = 3
ph7  = 1
ph3  = 1
ph6  = 1
```

ph8 = 1 3 2 0 2 0 3 1 3 1 0 2 0 2 1 3

ph16 = 1

ph10 = 0 2

ph2 = 0

ph4 = 0 0 2 2

ph5 = 0

ph12 = 0

ph20 = 0 0 0 0 2 2 2 2

ph15 = 1

ph13 = 0

ph14 = 0

ph31 = 0 2 2 0 2 0 0 2

REFERENCE

(1) Nimerovsky, E.; Najbauer, E. E.; Movellan, K. T.; Xue, K.; Becker, S.; Andreas, L. B. Modest Offset Difference Internuclear Selective Transfer via Homonuclear Dipolar Coupling. *J. Phys. Chem. Lett.* **2022**, *13* (6), 1540–1546. https://doi.org/10.1021/acs.jpclett.1c03871.

(2) Mehring, M. *Principles of High Resolution NMR in Solids*, 2nd ed.; Springer-Verlag: Berlin Heidelberg, 1983. https://doi.org/10.1007/978-3-642-68756-3.

(3) Haeberlen, U.; Waugh, J. S. Coherent Averaging Effects in Magnetic Resonance. *Phys. Rev.* **1968**, *175* (2), 453–467. https://doi.org/10.1103/PhysRev.175.453.

(4) Olejniczak, E. T.; Vega, S.; Griffin, R. G. Multiple Pulse NMR in Rotating Solids. *J. Chem. Phys.* **1984**, *81* (11), 4804–4817. https://doi.org/10.1063/1.447506.

(5) Lee, J.-S.; Regatte, R. R.; Jerschow, A. Selective Detection of Ordered Sodium Signals by a Jump-and-Return Pulse Sequence. *J. Magn. Reson.* **2009**, *200* (1), 126–129. https://doi.org/10.1016/j.jmr.2009.06.015.

(6) Nimerovsky, E.; Ilott, A. J.; Jerschow, A. Low-Power Suppression of Fast-Motion Spin 3/2 Signals. *J. Magn. Reson.* **2016**, *272*, 129–140. https://doi.org/10.1016/j.jmr.2016.09.007.

(7) Vega, S. Fictitious Spin 1/2 Operator Formalism for Multiple Quantum NMR. *J. Chem. Phys.* **1978**, *68* (12), 5518–5527. https://doi.org/10.1063/1.435679.

(8) Bayro, M. J.; Huber, M.; Ramachandran, R.; Davenport, T. C.; Meier, B. H.; Ernst, M.; Griffin, R. G. Dipolar Truncation in Magic-Angle Spinning NMR Recoupling Experiments. *J. Chem. Phys.* **2009**, *130* (11), 114506. https://doi.org/10.1063/1.3089370.

(9) Andreas, L. B.; Reese, M.; Eddy, M. T.; Gelev, V.; Ni, Q. Z.; Miller, E. A.; Emsley, L.; Pintacuda, G.; Chou, J. J.; Griffin, R. G. Structure and Mechanism of the Influenza A

M218–60 Dimer of Dimers. *J. Am. Chem. Soc.* **2015**, *137* (47), 14877–14886. https://doi.org/10.1021/jacs.5b04802.
(10) Nimerovsky, E.; Goldbourt, A. Insights into the Spin Dynamics of a Large Anisotropy Spin Subjected to Long-Pulse Irradiation under a Modified REDOR Experiment. *J. Magn. Reson.* **2012**, *225*, 130–141. https://doi.org/10.1016/j.jmr.2012.09.015.
(11) Schnell, J. R.; Chou, J. J. Structure and Mechanism of the M2 Proton Channel of Influenza A Virus. *Nature* **2008**, *451* (7178), 591–595. https://doi.org/10.1038/nature06531.
(12) Andreas, L. B.; Eddy, M. T.; Pielak, R. M.; Chou, J.; Griffin, R. G. Magic Angle Spinning NMR Investigation of Influenza A M218−60: Support for an Allosteric Mechanism of Inhibition. *J. Am. Chem. Soc.* **2010**, *132* (32), 10958–10960. https://doi.org/10.1021/ja101537p.
(13) Thakur, R. S.; Kurur, N. D.; Madhu, P. K. Swept-Frequency Two-Pulse Phase Modulation for Heteronuclear Dipolar Decoupling in Solid-State NMR. *Chem. Phys. Lett.* **2006**, *426* (4), 459–463. https://doi.org/10.1016/j.cplett.2006.06.007.
(14) Zhou, D. H.; Rienstra, C. M. High-Performance Solvent Suppression for Proton Detected Solid-State NMR. *J. Magn. Reson.* **2008**, *192* (1), 167–172. https://doi.org/10.1016/j.jmr.2008.01.012.
(15) Shaka, A. J.; Keeler, J.; Frenkiel, T.; Freeman, R. An Improved Sequence for Broadband Decoupling: WALTZ-16. *J. Magn. Reson. 1969* **1983**, *52* (2), 335–338. https://doi.org/10.1016/0022-2364(83)90207-X.
(16) Gullion, T.; Baker, D. B.; Conradi, M. S. New, Compensated Carr-Purcell Sequences. *J. Magn. Reson. 1969* **1990**, *89* (3), 479–484. https://doi.org/10.1016/0022-2364(90)90331-3.
